# Doubly robust estimators for generalizing treatment effects on survival outcomes from randomized controlled trials to a target population

**DOI:** 10.1515/jci-2022-0004

**Published:** 2022-12-09

**Authors:** Dasom Lee, Shu Yang, Xiaofei Wang

**Affiliations:** Department of Statistics, North Carolina State University, Raleigh, NC 27695, United States; Department of Statistics, North Carolina State University, Raleigh, NC 27695, United States; Department of Biostatistics and Bioinformatics, Duke University, Durham, NC 27708, United States

**Keywords:** causal inference, data integration, generalizability, survival analysis, semiparametric efficiency, 62D20, 62N02

## Abstract

In the presence of heterogeneity between the randomized controlled trial (RCT) participants and the target population, evaluating the treatment effect solely based on the RCT often leads to biased quantification of the real-world treatment effect. To address the problem of lack of generalizability for the treatment effect estimated by the RCT sample, we leverage observational studies with large samples that are representative of the target population. This article concerns evaluating treatment effects on survival outcomes for a target population and considers a broad class of estimands that are functionals of treatment-specific survival functions, including differences in survival probability and restricted mean survival times. Motivated by two intuitive but distinct approaches, i.e., imputation based on survival outcome regression and weighting based on inverse probability of sampling, censoring, and treatment assignment, we propose a semiparametric estimator through the guidance of the efficient influence function. The proposed estimator is doubly robust in the sense that it is consistent for the target population estimands if either the survival model or the weighting model is correctly specified and is locally efficient when both are correct. In addition, as an alternative to parametric estimation, we employ the nonparametric method of sieves for flexible and robust estimation of the nuisance functions and show that the resulting estimator retains the root-*n* consistency and efficiency, the so-called rate-double robustness. Simulation studies confirm the theoretical properties of the proposed estimator and show that it outperforms competitors. We apply the proposed method to estimate the effect of adjuvant chemotherapy on survival in patients with early-stage resected non-small cell lung cancer.

## 1 Introduction

In clinical trials or biomedical studies, time-to-event or survival endpoints, such as the time from treatment initiation to death, have been commonly used to evaluate the treatment effect. When estimating the treatment effect, randomized controlled trials (RCTs) are regarded as the gold standard since randomization reduces the effect of confounding variables. However, RCTs often suffer from a lack of generalizability or external validity. Specifically, due to restrictive inclusion and exclusion criteria for enrollment or additional concerns from patients and physicians, RCTs often do not recruit enough participants who represent the real-world patient population, resulting in the covariate distribution of the RCT sample being different from that of the target real-world population. In the presence of such heterogeneity, evaluating the treatment effect based solely on the RCT sample leads to biased quantification of the real-world treatment effect. As a complement of the RCT sample, observational studies have been widely used in comparative effectiveness research, as large samples that are representative of the target population can be studied at a relatively low cost.

Several recent works have proposed integrative methods to generalize findings from the RCT to the target population by leveraging observational studies [1-5]. Most existing methods focus on directly modeling the probability of participating in the trial, i.e., the sampling score that is analogous to the treatment propensity score. A widely used approach involves inverse probability of sampling weighting [IPSW; 1,6], which can be used to estimate weight-adjusted survival curves [7,8]. However, these IPSW-based estimators are unstable under extreme sampling scores. Alternatively, Lee et al. [5] proposed calibration weighting that enforces covariate balance between the RCT and observational study (OS) without explicitly modeling the sampling score. Recently, Colnet et al. [9] provided a comprehensive review of various novel methods combining complementary features of RCTs and observational studies. However, most of these methods focus on continuous and binary outcomes, and generalization of the findings from RCTs for survival outcomes to the target population is less actively studied.

In this article, we consider a broad class of estimands defined as a functional of treatment-specific survival functions, including differences in survival probability at landmark times and restricted mean survival time (RMST). Various estimators can be constructed to adjust for the unrepresentativeness or selection bias of the RCT sample. One approach relies on fitting conditional survival outcome models and then averaging over the covariate distribution of the observational sample, similar to Chen and Tsiatis [10]. Another common approach is to use weighting [7,11] to adjust for the imbalance between the RCT sample and the observational sample. Instead of direct modeling of sampling score as in IPSW, one can consider the more stable approach that calibrates covariate distributions between the RCT and the observational sample [5]. Motivated by these two intuitive but distinct approaches, we propose improved estimators for survival outcomes under the guidance of the efficient influence function (EIF), which involve survival outcome regression (OR) and weighting based on inverse probability of treatment, censoring, and sampling, simultaneously. The proposed estimator is doubly robust in the sense that it is consistent for the target population estimand if either the survival model or the weighting model is correctly specified, and is locally efficient when both are correct. In addition, to cope with possible misspecification of nuisance functions, we consider the method of sieves [12], which adds great flexibility and robustness to the proposed estimators, meanwhile retaining the root-*n* consistency.

The remainder of this article is organized as follows. In Section 2, we formalize the basic causal inference framework for survival outcomes. In Section 3, we introduce two direct estimators based on identification formulas, and in Section 4, we propose improved estimators and describe the corresponding asymptotic properties. The finite sample performance of the proposed estimators is assessed via simulation studies in Section 5. Applying the proposed estimators, we analyze the effect of adjuvant chemotherapy on the survival of lung cancer patients with data from an RCT and an OS in Section 6. Section 7 presents the discussion and concluding remarks. All proofs are provided in the Appendix.

## 2 Estimands, observed data, and assumptions

Suppose we are interested in comparing the effectiveness of two treatments. Let *A* be the binary treatment assignment, *A* ∈ {0, 1}. Following the potential outcomes framework [13,14], let *T*^*a*^ be the potential survival time if a subject received the treatment *A* = *a*, and *S*_*a*_(*t*) and *λ*_*a*_(*t*) be the corresponding survival and hazard functions, i.e., *S*_*a*_(*t*) = *P*(*T*^*a*^ ≥ *t*) and $\lambda_{a}(t)={\text{lim}}_{h\to 0}h^{-1}P(t\leq T^{a}\leq t+h)∕P(T^{a}\geq t)$. Under the proportional hazards assumption, a widely used measure to characterize the treatment effect is hazard ratio (HR), i.e., *λ*_1_(*t*)/*λ*_0_(*t*) being a constant. However, the interpretation of HRs is challenging especially when the proportionality assumption is violated [15,16].

Alternatively, we define the average treatment effect (ATE) measure *θ*_*τ*_ as a function of treatment-specific survival curves, *θ*_*τ*_ = Ψ_*τ*_(*S*_1_(*t*), *S*_0_(*t*)), where *τ* is a prespecified constant. This formulation of the ATE includes a broad class of estimands that are favored in survival analysis [17]. For example, *θ*_*τ*_ = *S*_1_(*τ*) − *S*_0_(*τ*) is a simple survival difference at a fixed time *τ*, and $\theta_{\tau }=\int_{0}^{\tau }\{S_{1}(t)-S_{0}(t)\}dt$ is the RMST difference up to *τ*. The ratio of restricted mean time loss and the difference of the median survival can also be represented with the appropriate choice of Ψ_*τ*_(·).

**Figure 1:**
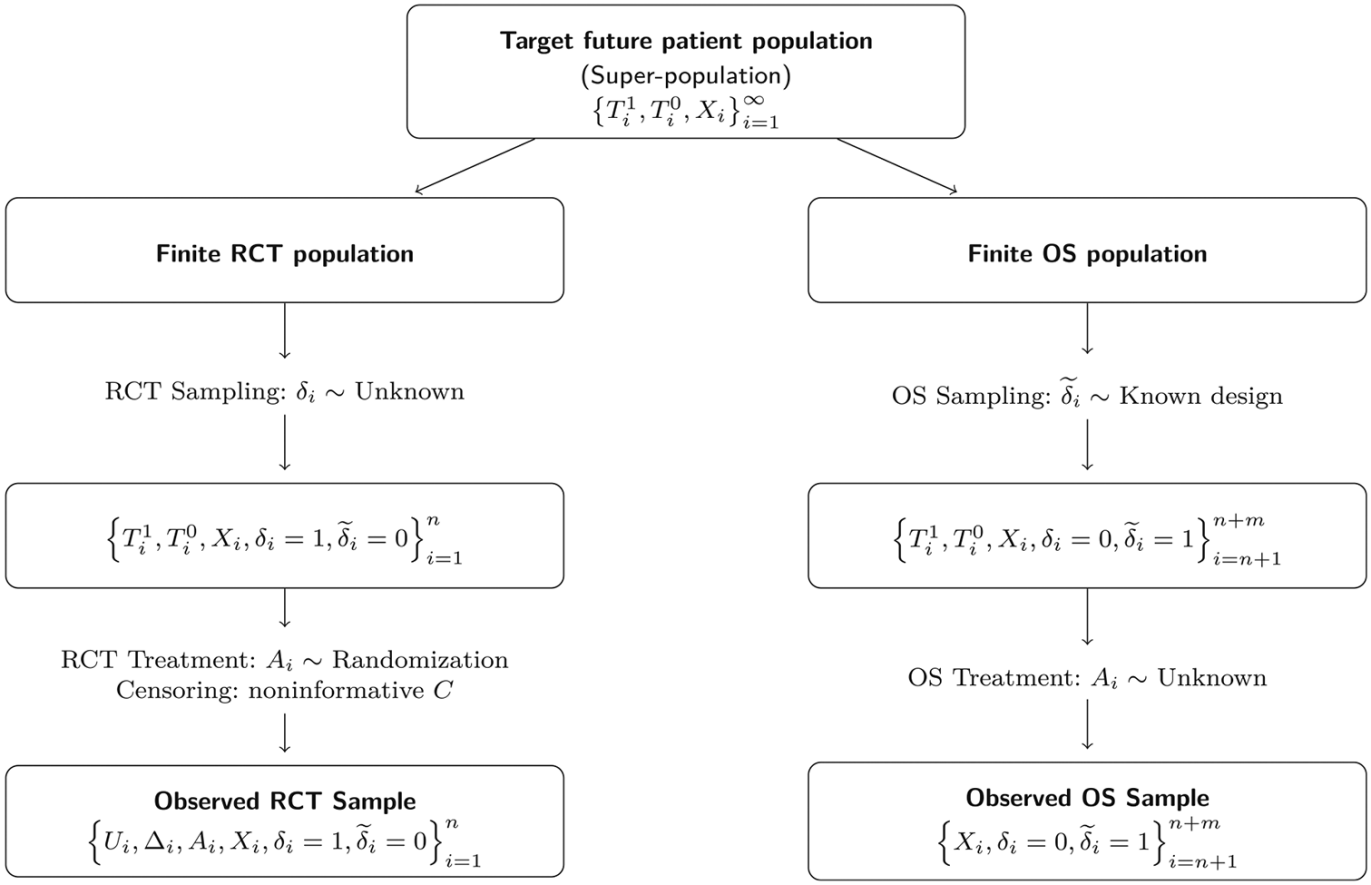
Illustration of the data structure of the RCT sample and the OS sample within the target super-population framework.

Under the stable unit treatment value assumption, the survival time *T* is the realization of the potential outcomes, i.e., *T* = *T*^1^*A* + *T*^0^(1 − *A*). Let *C* be the censoring time. In the presence of right censoring, the survival time *T* is not observed for all subjects; instead, we observe *U* = *T* ∧ *C* and Δ = *I*(*T* ≤ *C*), where ∧ represents the minimum of two values, and *I*(·) is an indicator function. Let *X* be a *p*-dimensional vector of pre-treatment covariates. Also, let *δ* denotes the binary indicator of RCT participation, and let $\tilde{\delta }$ denotes the binary indicator of OS participation. We consider a super-population framework assuming that an RCT sample of size *n* and an observational sample of size *m* are sampled from the target population. From the RCT sample, we observe $\left\{U_{i},\Delta_{i},A_{i},X_{i},\delta_{i}=1,{\tilde{\delta }}_{i}=0\right\}$ from *i* = 1, …, *n* independent and identically distributed subjects. For the observational sample, it is common that only the covariates information is available, i.e., $\left\{X_{i},\delta_{i}=0,{\tilde{\delta }}_{i}=1\right\}$ from *i* = *n* + 1,…, *n* + *m* independent and identically distributed subjects. The sampling mechanism and data structure are illustrated in Figure 1. We assume independence between the RCT and the observational sample, which holds if two separate studies are conducted by independent researchers, the target patient population is sufficiently large, or the patients are enrolled in two separate time periods.

Let *S*_*a*_(*t*, *X*) = *S*(*t*∣*X*, *A* = *a*, *δ* = 1) be the treatment-specific conditional survival curves for *a*, *δ* ∈ {0, 1}. Also, define the treatment propensity score *π*_*A*_(*X*) = *P*(*A* = 1∣*X*, *δ* = 1) and the sampling score *π*_*δ*_(*X*) = *P*(*δ* = 1∣*X*). To identify the ATE from the observed data, we make the following assumptions:

**Assumption 1.** (Ignorability and positivity of trial treatment assignment).

(i) {*T*^0^, *T*^1^} ⫫ *A*∣(*X*, *δ* = 1); and (ii) 0 < *π*_*A*_(*X*) < 1 with probability 1.

**Assumption 2.** (Conditional survival exchangeablity and positivity of trial participation).

(i) *S*_*a*_(*t*, *X*) = P(T^*a*^ > *t*∣*X*), *a* ∈ {0, 1}; and (ii) *π*_*δ*_(*X*) > 0 with probability 1.

**Assumption 3.** (Noninformative censoring conditional on covariates and treatment)

{*T*^1^, *T*^0^} ⫫ *C*∣(*X*, *A*, *δ* = 1), which also implies *T* ⫫ *C*∣(*X*, *A*, *δ* = 1).

Assumptions 1-3 are not testable in general, and their plausibility should be justified based on subject matter knowledge in practice. Assumption 1 holds in the RCT by default. Assumption 2 (i) is plausible if all information related to the trial participation and the outcome is captured in the data at hand. This assumption is weaker than the ignorablility of the trial participation assumption, i.e., {*T*^0^, *T*^1^} ⫫ *δ*∣*X*. The relationship between Assumption 2 (i) and its stronger version is analogous to that described in the study by Dahabreh et al. [4] in the context of continuous and binary outcomes. Assumption 2 (ii) implies that the absence of patient characteristics prevent from participating in the trial [5]. Assumption 3 is commonly made in survival analysis [10,18,19]. This assumption is weaker than the conditional independence assumption of the censoring and survival time given only on the treatment [20].

The covariate distribution of the RCT sample *f*(*X*∣*δ* = 1) may not be representative of that of the target population *f*(*X*) due to restrictive trial enrollment criteria, but the covariate distribution of the observational sample $f(X|\tilde{\delta }=1)$ is often representative of *f*(*X*) due to the real-world data collection mechanism. In particular, if the observational sample is a simple random sample of the target population, then $f(X|\tilde{\delta }=1)=f(X)$. More generally, the observational sample can be selected under complex sampling designs. To accommodate such scenarios, we can define *d* as the known design weight for the observational sample.

**Assumption 4.** (The known design weight for the observational sample). The observational sample design weight $d=1∕P(\tilde{\delta }=1|X)$ is known.

Assumption 4 is commonly assumed in the survey sampling literature. On the basis of Assumption 4, the design-weighted observational sample is representative of the target population. In an OS with simple random sampling, *d* = *N* / *m*, where *N* is the target population size.

Under the aforementioned assumptions, the ATE *θ*_*τ*_, or *S*_*a*_(*t*), *a* ∈ {0, 1} sufficiently, is identified based on the observed data. We consider two identification formulas. Let *Y*(*t*) = *I*(*U* ≥ *t*) and define the conditional censoring model $S_{a}^{C}(t,X)=P(C>t|X,A=a,\delta =1)$. One identification formula is based on the conditional survival curves, i.e.,

(1) $$S_{a}(t)=E\{\tilde{\delta }dS_{a}(t,X)\},\;\;S_{a}(t,X)=E\{I(T\geq t)|X,A=a,\delta =1,C\geq t\},$$

which can be called the OS-design-weighted G-computation formula. Note that if the population covariate distribution is available, $E\{S_{a}(t,X)\}$ is the G-computation formula for *S*_*a*_(*t*). The other identification formula is based on the inverse probability weighting (IPW) approach for the marginal survival curves, i.e.,

(2) $$S_{a}(t)=E\left[\frac{\delta }{\pi_{\delta }(X)}\frac{I(A=a)}{\{\pi_{A}\{X\}{\}}^{a}\{1-\pi_{A}(X){\}}^{1-a}}\frac{Y(t)}{S_{a}^{C}(t,X)}\right],$$

for *a* ∈ {0, 1}. The two identification formulas in (1) and (2) motivate the estimators in the following section, depending on different components of the observed data likelihood.

## 3 Two direct estimators based on identification formulas

### 3.1 Outcome regression

On the basis of the identification formula (1), the treatment-specific conditional survival curve can be modeled and fitted based on the observed data, e.g., using the widely used Cox regression model for the survival outcome. A treatment-specific conditional hazard function at time *t* given covariate *X*_*i*_ is defined as follows:

(3) $$\lambda_{ai}(t)\equiv \lambda_{a}(t|X_{i})=\lambda_{a0}(t)\;\text{exp}(\beta_{a}^{T}X_{i}),$$

where *λ*_*a*0_(*t*) is a treatment-specific baseline hazard function, for *a* ∈ {0, 1}, *i* = 1, …, *n*. Following standard survival analysis techniques, *β*_*a*_ can be estimated as a solution to the partial likelihood score equation, and the baseline cumulative hazard $\Lambda_{a0}(t)\equiv \int_{0}^{t}\lambda_{a0}(u)$ can be estimated by the Breslow [21] estimator:

$${\hat{\Lambda }}_{a0}(t)=\overset{\;t}{\underset{0\;\;}{\int }}\frac{\sum_{i=1}^{N}\delta_{i}A_{ai}dN_{i}(u)}{\sum_{i=1}^{N}\delta_{i}A_{ai}\;\text{exp}({\hat{\beta }}_{a}^{{}^{T}}X_{i})Y_{i}(u)},$$

where *A*_*ai*_ = *I*(*A*_*i*_ = *a*), *N*_*i*_(*u*) = *I*(*U*_*i*_ ≤ *u*, Δ_*i*_ = 1), and *Y*_*i*_(*u*) = *I*(*U*_*i*_ ≥ *u*). The survival model in (3) does not imply that the marginal HR of the potential survival outcomes under *a* = 1 and *a* = 0, i.e., *λ*_10_(*t*)/*λ*_00_(*t*), is a constant and thus is not restrictive. Other survival models can be considered, including the additive hazards model [22,23].

Chen and Tsiatis [10] proposed a method that accounts for imbalances between treatment groups by first estimating the conditional treatment effect given *X* and then averaging the effect over *X* across both treatment groups. A similar approach can be applied to balance the covariate distribution between the RCT sample and the observational sample by first estimating the treatment-specific survival curve conditional on *f*(*X*∣*δ* = 1) under model (3), i.e., ${\hat{S}}_{a}(t,X_{i})=\text{exp}\{-{\hat{\Lambda }}_{ai}(t)\}=\text{exp}\{-{\hat{\Lambda }}_{a0}(t)\;\text{exp}({\hat{\beta }}_{a}^{{}^{T}}X_{i})\}$, and then applying the design-weighted averaging over $f(X|\tilde{\delta }=1)$. The resulting OR estimator of the marginal treatment-effect survival curve is

(4) $${\hat{S}}_{a}^{{}^{\text{OR}}}(t)={\left(\sum_{i=1}^{N}{\tilde{\delta }}_{i}d_{i}\right)}^{-1}\sum_{i=1}^{N}{\tilde{\delta }}_{i}d_{i}e^{-{\hat{\Lambda }}_{ai}(t)},$$

for *a* ∈ {0, 1}, and the corresponding ATE estimator is defined as ${\hat{\theta }}_{\tau }^{\;{}^{OR}}=\Psi_{\tau }({\hat{S}}_{1}^{\;{}^{\text{OR}}}(t),{\hat{S}}_{0}^{\;{}^{\text{OR}}}(t))$. The OR estimator is consistent when the survival model (3) is correctly specified.

### 3.2 Inverse probability and calibration weighting

We can construct the estimator of the marginal treatment-specific survival curve based on the identification formula in (2) that involves sampling score, treatment propensity score, and censoring probability. This approach can be viewed as a combination of IPSW, inverse probability of treatment weighting (IPTW), and inverse probability of censoring weighting (IPCW). The weighting estimator requires positing models for the three probabilities and estimating them.

First, we consider the estimation of the sampling score. As the covariate distribution of the RCT sample is different from that of the target population in general, the estimated ATE based only on the RCT sample can be biased. A widely used approach to account for this selection bias is IPSW, i.e., weighting the RCT sample by the inverse probability of trial participation over that of OS participation, to adjust for differences in covariate distribution between the trial sample and the population. Specifically, *π*_*δ*_(*X*) can be modeled as *π*_*δ*_(*X*) = {*ω*_IPSW_(*X*)}^−1^, where $\omega_{\text{IPSW}}(X)=P(\tilde{\delta }=1|\delta +\tilde{\delta }=1,X)∕P(\delta =1|\delta +\tilde{\delta }=1,X)$. One can plug in $\{{\hat{\omega }}_{\text{IPSW}}(X){\}}^{-1}$ for *π*_*δ*_(*X*) in (2) using the common logistic regression model. However, the IPSW method requires the sampling score model to be correctly specified; it also could be highly unstable if $P(\delta =1|\delta +\tilde{\delta }=1,X)$ is close to zero for some *X*.

Instead of direct estimating the sampling scores, Lee et al. [5] proposed the calibration weighting approach to reduce the selection bias in the trial-based estimator, which is analogous to the entropy balancing method by Hainmueller [24] and more stable than the IPSW method. The basic idea is that subjects in the RCT sample are calibrated to the observational sample, so that after calibration, the covariate distribution of the RCT sample empirically matches that of the target population. The calibration weighting approach is based on the idea that for any ***g***(*X*), the following identity holds,

(5) $$E\left\{\frac{\delta }{\pi_{\delta }(X)}g(X)\right\}=E\{\tilde{\delta }dg(X)\}=E\{g(X)\},$$

where ***g***(*X*) is a function of *X* to be calibrated, e.g., the moments or any nonlinear transformations.

The calibration weights *ω*_*i*_ are obtained by solving the optimization problem:

(6) $$\underset{𝒲}{\text{min}}\sum_{i=1}^{n}\omega_{i}\;\log\;\omega_{i},\;\;\text{subject to}\;\omega_{i}\geq 0,\;\;\forall i,\;\;\sum_{i=1}^{n}\omega_{i}=1,\;\;\text{and}\;\;\sum_{i=1}^{N}\delta_{i}\omega_{i}g(X_{i})=\tilde{g},$$

where $𝒲=\{w_{i}\;:\delta_{i}=1\}$. The last constraint is the empirical representation of (5), where $\tilde{g}=\sum_{i=1}^{N}{\tilde{\delta }}_{i}d_{i}g(X_{i})∕\sum_{i=1}^{N}{\tilde{\delta }}_{i}d_{i}$ is a consistent estimator of E{g(X)} from the observational sample. Minimizing the negative entropy function of the calibration weights in (6) enforces the weights to be as close to one another as possible, which reduces the variability due to heterogeneous weights. By using the Lagrange multiplier ***λ***, the objective function of this convex optimization problem becomes $L(\lambda ,𝒲)=\sum_{i=1}^{n}\omega_{i}\;\log\omega_{i}-\lambda^{T}\{\sum_{i=1}^{n}\omega_{i}g(X_{i})-\tilde{g}\}$. The estimated calibration weights are $\hat{\omega_{i}}=\omega (X_{i};\;\hat{\lambda })=\text{exp}\{{\hat{\lambda }}^{T}g(X_{i})\}∕[\sum_{i=1}^{n}\text{exp}\{{\hat{\lambda }}^{T}g(X_{i})\}]$, where $\hat{\lambda }$ solves

(7) $$U(\lambda )=\sum_{i=1}^{n}\;\text{exp}\{\lambda^{T}g(X_{i})\}\{g(X_{i})-\tilde{g}\}=0.$$

Under the loglinear sampling score model, the calibration weights from the objective function (6) have the same functional form as inverse probability of sampling score weights asymptotically, resulting in the direct correspondence between the calibration weight and the sampling score in that ${\hat{\omega }}_{i}-\{N{\hat{\pi }}_{\delta }(X_{i}){\}}^{-1}\overset{p}{\to }0$, as *n* → ∞ [5]. Following that, we posit the loglinear sampling score model,

(8) $$\pi_{\delta }(X)=\text{exp}\{\eta_{0}^{T}g(X)\},\;\;\text{for some}\;\eta_{0}.$$

Lee et al. [5] showed that $\hat{\lambda }$ is equivalent to $-\hat{\eta }$, where ${\hat{\pi }}_{\delta }(X)=\pi_{\delta }(X;\;\hat{\eta })=\text{exp}\{{\hat{\eta }}^{T}g(X)\}$. Accordingly, in the rest of the article, we represent the calibration weights using $\hat{\eta }$, i.e., ${\hat{\omega }}_{i}=\omega (X_{i};\;\hat{\eta })=\text{exp}\{-{\hat{\eta }}^{T}g(X_{i})\}∕[\sum_{i=1}^{n}\;\text{exp}\{-{\hat{\eta }}^{T}g(X_{i})\}]$. If one considers a logistic sampling score model instead, then other objective functions can be used, such as $\sum_{i=1}^{n}(\omega_{i}-1)\;\log(\omega_{i}-1)$, that corresponds to the weights with the same functional form as the inverse to a logistic probability of sampling [25,26]. However, the loglinear regression model in (8) is close to the logistic regression model when the proportion of the RCT sample in the target population is small.

With respect to treatment assignment, *π*_*A*_(*X*) is generally known for RCTs. However, several authors suggested estimating the treatment propensity score for the RCTs to increase the efficiency and account for the chance of imbalance of prognostic variables [e.g., 27,28]. We choose a logistic regression model for the treatment propensity score,

(9) $$\pi_{A}(X)=[1+\text{exp}\{-\rho_{0}^{T}g(X)\}{]}^{-1},\;\;\text{for some}\;\rho_{0},$$

and define ${\hat{\pi }}_{ai}=A_{i}\pi_{A}(X_{i};\;\hat{\rho })+(1-A_{i})\{1-\pi_{A}(X_{i};\;\hat{\rho })\}$. Estimating the propensity scores also broadens the scope of the current article to allow the generalization of the OS. Even though this article focuses on generalizing the trial findings where we only require a two-way balancing between the RCT and OS, a three-way balancing approach among the treated, the controlled, and the observational sample (e.g., [29]) can be useful to generalize the findings from the OS to its larger population with the estimation of *π*_*A*_.

Moreover, to account for right censoring *C*, we posit Cox proportional hazards model with conditional hazard

(10) $$\lambda^{C}(t|X,A=a)=\lambda_{a0}^{C}(t)\;\text{exp}(\gamma_{a}^{T}X),\;\;\text{for}\;a\in \{0,1\},$$

where the standard techniques as for the survival model in (3) can be used to estimate *γ*_*a*_ and $\Lambda_{a0}^{C}(t)\;\equiv \;\int_{0}^{t}\lambda_{a0}^{C}(u)du$.

Combining ${\hat{\omega }}_{i}$, ${\hat{\pi }}_{ai}$, and ${\hat{\Lambda }}_{ai}^{C}(t)={\hat{\Lambda }}_{a0}^{C}(t)\;\text{exp}({\hat{\gamma }}_{a}^{T}X_{i})$ estimated under the working models in (8), (9), and (10), respectively, we define the CW estimator of the marginal treatment-specific survival curve as follows:

(11) $${\hat{S}}_{a}^{{}^{CW}}(t)=\sum_{i=1}^{N}\delta_{i}{\hat{\omega }}_{i}\frac{A_{ai}}{{\hat{\pi }}_{ai}}e^{{\hat{\Lambda }}_{ai}^{C}(t)}Y_{i}(t),$$

where *A*_*ai*_ = *I*(*A*_*i*_ = *a*). The corresponding ATE estimator is ${\hat{\theta }}_{\tau }^{{}^{CW}}=\Psi_{\tau }({\hat{S}}_{1}^{{}^{CW}}(t),{\hat{S}}_{0}^{{}^{CW}}(t))$.

## 4 Improved estimators

### 4.1 Efficient influence function

Let $𝒪=(X,A,U,\Delta ,\delta ,\tilde{\delta })$ be one copy of the vector of *observed variables*. The OR estimator specified in (4) and the CW estimator specified in (11) use different components of the likelihood function f(𝒪). Specifically, the OR estimator is based on modeling *S*_*a*_(*t*, *X*) for a ∈ {0, 1}, and the CW estimator is based on modeling *π*_*δ*_(*X*), *π*_*a*_(*X*), and $S_{a}^{C}(t,X)$ for *a* ∈ {0, 1}. These estimators are singly robust in that they are consistent only under the correct survival OR model or the correct weighting models. A vast number of estimators can be constructed by combining these two estimators. In general, the question becomes how to obtain the most efficient estimator. Our approach is to derive the EIF [30] of *θ*_*τ*_ to construct semiparametrically efficient estimators. Such estimators also gain robustness to model misspecification as we show later.

We consider a class of influence functions of regular asymptotically linear (RAL) estimators of the treatment-specific survival curve *S*_*a*_(*t*). Define *A*_*a*_ = *I*(*A* = *a*) and *π*_*a*_(*X*) = *aπ*_*A*_(*X*) + (1 − *a*){1 − *π*_*A*_(*X*)}. Following Tsiatis [30], the class of observed data influence functions includes

(12) $$\varphi_{a}(t;𝒪)=\frac{\delta }{\pi_{\delta }(X)}\frac{A_{a}}{\pi_{a}(X)}\frac{Y(t)}{S_{a}^{C}(t,X)}-S_{a}(t)\;\;+\overset{\text{an arbitrary score of}\;A|(X,\delta =1)}{\overset{︷}{\frac{\delta }{\pi_{\delta }(X)}\{A-\pi_{A}(X)\}g_{a1}(X)}}+\overset{\text{an arbitrary score of}\;\delta |X}{\overset{︷}{\left\{\frac{\delta }{\pi_{\delta }(X)}-\tilde{\delta }d\right\}g_{a2}(X)}}\;\;+\;\overset{\text{an arbitrary score of}\;U,\Delta =0|(X,A,\delta =1)}{\overset{︷}{\frac{\delta }{\pi_{\delta }(X)}\frac{A_{a}}{\pi_{a}(X)}\overset{\;t}{\underset{0\;}{\int }}\frac{dM_{a}^{C}(u)}{S_{a}^{C}(u,X)}g_{a3}(u|A=a,X)}}$$

for arbitrary functions *g*_*a*1_(·), *g*_*a*2_(·), and *g*_*a*3_(·), where $M_{a}^{C}(u)=N_{a}^{C}(u)-\int_{0}^{u}\Lambda_{a}^{C}(s)ds$ is a Martingale with $N_{a}^{C}=A_{a}I(U\leq u,\Delta =0)$, and $\Lambda_{a}^{C}(s)$ is a cumulative hazard for censoring for *a* ∈ {0, 1}. The last three terms in (12) are mean-zero functions. According to the semiparametric theory [30], the EIF is the influence function in (12) with the smallest variance. The EIF can be derived by projecting the first term of (12) onto the orthogonal complement of the tangent space spanned by the scores of the nuisance functions, i.e., the last three terms. The RAL estimator with the EIF is a semiparametrically efficient estimator. The following theorem gives the EIF in the class of influence functions (12), with the proof given in the Appendix B.1.

**Theorem 1.** *Under* Assumptions 1-4, *the EIF for the treatment-specific survival curve is*

(13) $$\varphi_{a}^{\text{eff}}(t;𝒪)=\frac{\delta }{\pi_{\delta }(X)}\frac{A_{a}}{\pi_{a}(X)}\frac{Y(t)}{S_{a}^{C}(t,X)}-S_{a}(t)\;\;-\frac{\delta }{\pi_{\delta }(X)}\left\{\frac{A-\pi_{A}(X)}{\pi_{A}(X)}\right\}E\{I(T\geq t)|X,A=a,\delta =1\}\;\;-\left\{\frac{\delta }{\pi_{\delta }(X)}-\tilde{\delta }d\right\}E\{I(T\geq t)|X,A=a,\delta =1\}\;\;+\frac{\delta }{\pi_{\delta }(X)}\frac{A_{a}}{\pi_{a}(X)}\overset{\;t}{\underset{0\;}{\int }}\frac{dM_{a}^{C}(u)}{S_{a}^{C}(u,X)}E\{I(T\geq u)|X,A=a,\delta =1,U\geq u\}.$$

Many common treatment effect estimands are functionals of the treatment-specific survival curves, including the survival difference at a fixed time *τ*, the difference of RMSTs, the ratio of RMTLs, and the difference of *τ*th quantile of survivals. Their EIFs can be expressed in the form of a combination of weighted integrals of the EIF for treatment-specific survival curves [17]. To be specific, the EIF for *θ*_*τ*_ is

(14) $$\varphi_{\theta_{\tau }}^{\;\text{eff}}(𝒪)=\overset{\;\tau }{\underset{0\;}{\int }}\phi_{1}(t)\varphi_{1}^{\;\text{eff}}(t;\;𝒪)dt+\overset{\;\tau }{\underset{0\;}{\int }}\phi_{0}(t)\varphi_{0}^{\;\text{eff}}(t;\;𝒪)dt$$

for some functions *ϕ*_*a*_(·) that $E\{\phi_{a}(\cdot {)}^{2}\}<\infty$ (see Appendix A for details). We limit our interest to the estimands with such form of the EIF, which covers the broad class of estimators that are favored in survival analysis.

### 4.2 Augmented calibration weighting estimator

Motivated by Theorem 1, under the survival model in (3) and the weighting models specified in (8)-(10), we propose the following augmented CW (ACW) estimator of the treatment-specific survival curve,

(15) $${\hat{S}}_{a}^{{}^{\text{ACW}1}}(t)=\sum_{i=1}^{N}\delta_{i}{\hat{\omega }}_{i}\frac{A_{ai}}{{\hat{\pi }}_{ai}}e^{{\hat{\Lambda }}_{ai}^{C}(t)}Y_{i}(t)-\sum_{i=1}^{N}\delta_{i}{\hat{\omega }}_{i}\left(\frac{A_{ai}-{\hat{\pi }}_{ai}}{{\hat{\pi }}_{ai}}\right)e^{-{\hat{\Lambda }}_{ai}(t)}\;\;-\;\sum_{i=1}^{N}\left\{\delta_{i}{\hat{\omega }}_{i}-{\left(\sum_{i=1}^{N}{\tilde{\delta }}_{i}d_{i}\right)}^{-1}{\tilde{\delta }}_{i}d_{i}\right\}e^{-{\hat{\Lambda }}_{ai}(t)}+\sum_{i=1}^{N}\delta_{i}{\hat{\omega }}_{i}\frac{A_{ai}}{{\hat{\pi }}_{ai}}\overset{\;t}{\underset{0\;}{\int }}\frac{d{\hat{M}}_{ai}^{C}(u)}{e^{-{\hat{\Lambda }}_{ai}^{C}(u)}}\frac{e^{-{\hat{\Lambda }}_{ai}(t)}}{e^{-{\hat{\Lambda }}_{ai}(u)}}\;\;=\sum_{i=1}^{N}\delta_{i}{\hat{\omega }}_{i}\frac{A_{ai}}{{\hat{\pi }}_{ai}}e^{{\hat{\Lambda }}_{ai}^{C}(t)}Y_{i}(t)+\sum_{i=1}^{N}e^{-{\hat{\Lambda }}_{ai}(t)}\left[{\left(\sum_{i=1}^{N}{\tilde{\delta }}_{i}d_{i}\right)}^{-1}{\tilde{\delta }}_{i}d_{i}-\delta_{i}{\hat{\omega }}_{i}\frac{A_{ai}}{{\hat{\pi }}_{ai}}\left\{1-\overset{\;t}{\underset{0\;}{\int }}\{e^{{\hat{\Lambda }}_{ai}^{C}(u)+{\hat{\Lambda }}_{ai}(u)}\}d{\hat{M}}_{ai}^{C}(u)\right\}\right].$$

In addition, following the technique by Zhang and Schaubel [20] that represents the marginal cumulative hazard function $\Lambda_{a}(t)=\int_{0}^{t}-\{S_{a}(u){\}}^{-1}dS_{a}(u)$ by estimating the denominator and the numerator separately, we propose another ACW estimator,

(16) $${\hat{S}}_{a}^{{}^{\text{ACW}2}}(t)=\text{exp}\left\{-\overset{\;t}{\underset{0\;}{\int }}\frac{-d{\hat{S}}_{a}^{{}^{\text{ACW}1}}(u)}{{\hat{S}}_{a}^{{}^{\text{ACW}1}}(u)}\right\},$$

where

−dS^aACW1(u)=∑i=1Nδiω^iAaiπ^aieΛ^aiC(u)dNi(u)+∑i=1Ne−Λ^ai(u)dΛ^ai(u)[(∑i=1Nδ~idi)−1δ~idi]−[δiω^iAaiπ^ai{1−∫0u{eΛ^aiC(s)+Λ^ai(s)}dM^aiC(s)}]

estimates −d*S*_*a*_(*u*). Although ${\hat{S}}_{a}^{\;{}^{\text{ACW}1}}(t)$ and ${\hat{S}}_{a}^{\;{}^{\text{ACW}2}}(t)$ are asymptotically equivalent, simulation studies show that ${\hat{S}}_{a}^{\;{}^{\text{ACW}2}}(t)$ has better finite-sample performance. The proposed ACW estimators are similar to the estimators developed by Zhang and Schaubel [18] and Zhang et al. [19]. The difference between the proposed and their method is discussed in Section 7.

Similar to Yang et al. [17], we consider an asymptotic linear characterization of the ATE estimator ${\hat{\theta }}_{\tau }^{\;{}^{\text{ACW}}}=\Psi_{\tau }({\hat{S}}_{1}^{\;{}^{\text{ACW}}}(t),{\hat{S}}_{0}^{\;{}^{\text{ACW}}}(t))$ for both ACW1 and ACW2 estimators. That is, under mild regularity conditions,

(17) $${\hat{\theta }}_{\tau }^{\;{}^{\text{ACW}}}-\theta_{\tau }=\overset{\;\tau }{\underset{0\;}{\int }}\phi_{1}(t)\{{\hat{S}}_{1}^{\;{}^{\text{ACW}}}(t)-S_{1}(t)\}dt+\overset{\;\tau }{\underset{0\;}{\int }}\phi_{0}(t)\{{\hat{S}}_{0}^{\;{}^{\text{ACW}}}(t)-S_{0}(t)\}dt+o_{p}(N^{-1∕2}),$$

for bounded variation functions *ϕ*_*a*_(·) in (14). Under the asymptotic linear characterization, ${\hat{\theta }}_{\tau }^{\;{}^{\text{ACW}}}$ has the influence function $\varphi_{\theta_{\tau }}^{\;\text{eff}}(𝒪)$ (see Appendix B.2 for the proof).

Toward this end, the following theorem shows the local efficiency and asymptotic properties of the proposed ACW estimators of the ATE, i.e., ${\hat{\theta }}_{\tau }^{\;{}^{\text{ACW}1}}=\Psi_{\tau }({\hat{S}}_{1}^{\;{}^{\text{ACW}1}}(t),{\hat{S}}_{0}^{\;{}^{\text{ACW}1}}(t))$ and ${\hat{\theta }}_{\tau }^{\;{}^{\text{ACW}2}}=\Psi_{\tau }({\hat{S}}_{1}^{\;{}^{\text{ACW}2}}(t),{\hat{S}}_{0}^{\;{}^{\text{ACW}2}}(t))$.

**Theorem 2.** *Under* Assumptions 1-4, *if either the survival model in* (3) *is correctly specified or the weighting models, i.e., the sampling score model* (8), *the treatment propensity score model* (9), *and the censoring model* (10), *are correctly specified, under regularity conditions*, ${\hat{\theta }}_{\tau }^{\;{}^{\text{ACW}1}}$ *and* ${\hat{\theta }}_{\tau }^{\;{}^{\text{ACW}2}}$ *are consistent for θ_τ_, and* $N^{1∕2}({\hat{\theta }}_{\tau }^{\;{}^{\text{ACW}1}}-\theta_{\tau })$ *and* $N^{1∕2}({\hat{\theta }}_{\tau }^{\;{}^{\text{ACW}2}}-\theta_{\tau })$ *are asymptotically normal with mean zero and variance* $E(\varsigma_{1}^{2})$ *and* $E(\varsigma_{2}^{2})$. *Moreover, if all working models in* (3) *and* (8)-(10) *are correctly specified*, ${\hat{\theta }}_{\tau }^{\;{}^{\text{ACW}1}}$ *and* ${\hat{\theta }}_{\tau }^{\;{}^{\text{ACW}2}}$ *are locally efficient, i.e.*, $\varsigma_{1}=\varsigma_{2}=\varphi_{\theta_{\tau }}^{\;\text{eff}}(𝒪)$ *as n* → ∞.

The proof of Theorem 2 and details of $\varsigma_{1}^{2}$ and $\varsigma_{2}^{2}$ are provided in Appendix B.3. For a straightforward procedure of variance estimation, a nonparametric bootstrap method can be used. Specifically, we draw *B* bootstrap samples from both the RCT and the observational sample, respectively, and then for each resampled pair, we obtain a replicate of the ACW estimator; the sample variance of the *B* bootstrap replicates is the variance of the ACW estimator.

The proposed ACW estimators depend on the parametric estimation of nuisance functions. Alternatively, we can consider a flexible nonparametric approach without the parametric assumption, which is often unrealistic in complex problems in practice. The asymptotic behavior of the ACW estimators with the nonparametric estimation of nuisance functions can be characterized by the empirical process perspective. Suppose that the posited nuisance models are consistent, i.e., $\left\|\pi_{\delta }(X;\;\hat{\eta })-\pi_{\delta }(X)\|=o_{p}(1\right)$, $\left\|\pi_{A}(X;\;\hat{\rho })-\pi_{A}(X)\|=o_{p}(1\right)$, and $\left\|S_{a}(t,X;{\hat{\beta }}_{a})-S_{a}(t,X)\|=o_{p}(1),\;\;\|S_{a}^{C}(t,X;\;{\hat{\gamma }}_{a})-S_{a}^{C}(t,X)\|=o_{p}(1\right)$, for *a* ∈ {0, 1}. Also, suppose that the weighting functions $\pi_{\delta }(X;\;\hat{\eta })$, $\pi_{A}(X;\;\hat{\rho })$, and $S_{a}^{C}(u,X;\;{\hat{\gamma }}_{a})$ are bounded away from zero. Then, we have the effect of the estimated nuisance functions in ${\hat{\theta }}_{\tau }^{\;{}^{\text{ACW}}}-\theta_{\tau }$ bounded above by ∑a=01∫0τϕa(t){‖πδ(X;η^)−πδ(X)‖⋅‖Sa(t,X;β^a)−Sa(t,X)‖+‖πA(X;ρ^)−πA(X)‖⋅‖Sa(t,X;β^a)−Sa(t,X)+{P∫0t‖dMaC(u,X;γ^a)‖⋅‖Sa(u,X)−1Sa(t,X)−Sa(u,X;β^a)−1Sa(t,X;β^a)‖} up to a multiplicative constant, where P is a true measure such that Pf(𝒪)=∫f(𝒪)dP and ∥·∥ is *L*_2_ norm. If each term of the bound is of rate *o*_*p*_(*n*^−1/2^), then the effect of nuisance function estimations are asymptotically negligible. This statement is formalized in the following theorem.

**Theorem 3.** *Suppose* Assumptions 1-4 *hold. Let* $S_{a}(t,X;{\hat{\beta }}_{a})$ *be general semiparametric and nonparametric models for S*_*a*_(*t*, *X*), *and let* $\pi_{\delta }(X;\;\hat{\eta })$, $\pi_{A}(X;\;\hat{\rho })$, *and* $S_{a}^{C}(t,X;\;{\hat{\gamma }}_{a})$ *be general semiparametric models for π_δ_*(*X*), *π_A_*(*X*), *and* $S_{a}^{C}(t,X)$, *respectively, for a* ∈ {0, 1}. *Suppose the following conditions hold*:

(C1) $$\|S_{a}(t,X;{\hat{\beta }}_{a})-S_{a}(t,X)\|=o_{p}(1),\;\;\;\;\;\;\|\pi_{\delta }(X;\;\hat{\eta })-\pi_{\delta }(X)\|=o_{p}(1),\;\;\;\;\;\;\|\pi_{A}(X;\;\hat{\rho })-\pi_{A}(X)\|=o_{p}(1),\;\;\|S_{a}^{C}(t,X;\;{\hat{\gamma }}_{a})-S_{a}^{C}(t,X)\|=o_{p}(1);$$

(C2) $$0<c_{1}\leq \pi_{\delta }(X;\;\hat{\eta }),\pi_{A}(X;\;\hat{\rho }),S_{a}^{C}(u,X;\;{\hat{\gamma }}_{a})\leq c_{2}\leq 1\;for\;some\;c_{1},c_{2};$$

(C3) $$\|\pi_{\delta }(X;\;\hat{\eta })-\pi_{\delta }(X)\|\cdot \|S_{a}(t,X;{\hat{\beta }}_{a})-S_{a}(t,X)\|=o_{p}(n^{-1∕2}),\;\;\|\pi_{A}(X;\;\hat{\rho })-\pi_{A}(X)\|\cdot \|S_{a}(t,X;{\hat{\beta }}_{a})-S_{a}(t,X)\|=o_{p}(n^{-1∕2}),$$

$$P\overset{\;t}{\underset{0\;}{\int }}\|dM_{a}^{C}(u,X;\;{\hat{\gamma }}_{a})\|\cdot \|S_{a}(u,X{)}^{-1}S_{a}(t,X)-S_{a}(u,X;{\hat{\beta }}_{a}{)}^{-1}S_{a}(t,X;{\hat{\beta }}_{a})\|=o_{p}(n^{-1∕2}).$$

*Then*, ${\hat{\theta }}_{\tau }^{\;{}^{\text{ACW}1}}$ *and* ${\hat{\theta }}_{\tau }^{\;{}^{\text{ACW}2}}$ *are consistent estimators for θ_τ_ and achieve semiparametric efficiency*.

The proof of Theorem 3 is provided in Appendix B.4. To ensure the consistency of the nuisance function estimation with the convergence rate of the product as in (C3), we use the method of sieves in Section 4.3.

### 4.3 Robust estimation using the method of sieves with penalization

For a robust estimation of the ATE under possibly misspecified working models, we adopt the method of sieves [31,32], which enables flexible data-adaptive estimation of the survival curves and probability weights with root-n consistency [12]. We construct the sieves using the linear spans of power series [33], but other basis functions such as Fourier series or splines are applicable. Specifically, for a *p*-dimensional vector of non-negative integers *κ_k_* = (*κ*_*k*1_, …, *κ*_*kp*_), we consider a *K*-vector sieve basis functions ***g***(*X*) = {*g*_1_(*X*), …, *g*_*K*_(*X*)}^T^ = {*X*^*κ*_1_^ …, *X*^*κ*_*K*_^}^T^, where $X^{\kappa_{k}}=\prod_{l=1}^{p}X_{l}^{\kappa_{kl}}$ with $|\kappa_{k}|=\sum_{l=1}^{p}\kappa_{kl}$ non-decreasing in *k*, i.e., ∣*κ*_*k*_∣ ≤ ∣*κ*_*k*+1_∣. Under standard regularity conditions, the sieves approximation results in a consistent estimation of the survival curves and weighting probabilities with large *K* (see Lee et al. [5], supporting information). To facilitate the stable estimation and to control the variability of the estimators with large *K*, we consider the penalized estimation of the nuisance functions.

For the sampling score model *π*_*δ*_(*X*), the penalized sieves estimation is based on the dual problem of calibration that solves the estimating equation *U*(***λ***) in (7). Following the penalized estimating equation approach [34-36], we solve

$$U^{\varepsilon }(\lambda )=U(\lambda )-q_{\varepsilon }(|\lambda |)\;\text{sign}\;(\lambda )$$

for ***λ*** = (*λ*_1_, …, *λ*_*K*_)^T^, where *q*_*ε*_(∣***λ***∣) sign (***λ***) is the element-wise product of *q*_*ε*_(∣***λ***∣) = {*q*_*ε*_(∣*λ*_1_∣), …, *q*_*ε*_(∣*λ*_*K*_∣)}^T^ and sign (***λ***). We define *q*_*ε*_(*x*) = d*p*_*ε*_(*x*)/d*x* and specify *p*_*ε*_(*x*) to be the popular smoothly clipped absolute deviation (SCAD) penalty function [37], but different penalty functions such as adaptive lasso [38] and the minimax concave penalty [39] are also applicable. For the SCAD penalty, we have *q*_*ε*_(∣*λ*_*k*_∣) = *ε*[*I*(∣*λ*_*k*_∣ < *ε*) + {(*b* − 1)*ε*}^−1^{*bε* − ∣*λ*_*k*_∣)_+_}*I*(∣*λ*_*k*_∣ ≥ *ε*)], for *k* = 1, …, *K*, with *b* = 3.7 following the literature and the tuning parameter *ε* selected by cross validation.

For penalized sieves estimation of the survival outcome model *S*_*a*_(*t*, *X*), the standard penalization technique for the Cox PH model was used based on the partial likelihood. Specifically, we estimate *β*_*a*_ by solving

$$\text{arg}\max_{\beta_{a}\in R^{K}}\left[\sum_{r\in D}\delta_{r}A_{ar}\left\{\beta_{a}^{T}g(X_{r})-\log\left[\sum_{l\in R_{r}}\text{exp}\{\beta_{a}^{T}g(X_{l})\}\right]\right\}-\sum_{j=1}^{K}p_{\varepsilon }(|\beta_{aj}|)\right],$$

where *D* is the set of indices of the events, *R*_*r*_ is the set of indices of the patients at risk at time *t*_*r*_, with *t*_1_< … < *t*_*d*_ denoting the distinct event time, and *p*_*ε*_(·) is the SCAD penalty, for *a* ∈ {0, 1}. The penalized sieves estimation of the censoring model $S_{a}^{C}(t,X)$ can be adopted by analogy. Finally, for the penalized sieves estimation of the treatment propensity score model *π*_*A*_(*X*), we use the standard penalization approach for the binary data using the logit link with the SCAD penalty.

Under regularity conditions specified in Fan and Li [37] and Lee et al. [5], the penalized sieve estimators of the nuisance functions possess oracle properties and satisfy two conditions in Theorem 3. Thus, the resulting ACW estimators using the method of sieves achieve the root-n consistency and semiparametric efficiency.

## 5 Simulation studies

In this section, we conduct simulation studies to evaluate the finite-sample performance of the proposed estimators. We consider the target population of size *N* = 200,000 and covariates *X* = (*X*_1_, *X*_2_, *X*_3_)^T^, where each *X*_*i*_, *i* = 1, 2, 3 is generated from *N*(0, 1) and truncated at −4 and 4 to satisfy regularity conditions. An RCT sample of size *n* ~ 1,300 is selected from a hypothetical RCT eligible population with size *N*_1_ = 50,000. From the remaining *N*_2_ = 150,000 OS population, we randomly select a sample of size *m* = 5,000.

We consider the difference in RMST as the ATE, i.e., $\theta_{\tau }=\Psi_{\tau }(S_{1}(t),S_{0}(t))=\int_{0}^{\tau }\{S_{1}(t)-S_{0}(t)\}dt=\int_{0}^{\tau }S_{1}(t)dt-\int_{0}^{\tau }S_{0}(t)dt=\mu_{1,\tau }-\mu_{0,\tau }$, where we choose *τ* = 20. We compare the proposed CW and the ACW estimators with four other methods, the Naive estimator based only on the RCT sample, the ZS estimator, i.e., the estimator proposed by Zhang and Schaubel [18] based only on the RCT sample, the IPSW estimator using the normalized IPSW weight in (11), and the OR estimator specified in (4). For the ACW estimators, in addition to the original covariate vector *g*_1_(*X*) = (*X*_1_, *X*_2_, *X*_3_)^T^, we consider the penalized sieve estimation with calibration variables $g_{2}(X)=(X_{1},X_{2},X_{3},X_{1}X_{2},X_{1}X_{3},X_{2}X_{3},X_{1}^{2},X_{2}^{2},X_{3}^{2}{)}^{T}$, i.e., the extension of the basis functions in *g*_1_(*X*) to its second-order power series including all two-way interactions and quadratic terms. We use (S) to indicate the method of sieves. To assess the performance of these estimators under model misspecification, we consider four scenarios where (i) all four models in (3) and (8)-(10) are correct, (ii) only the survival outcome model (3) is correct, (iii) the outcome model is incorrect, but the other three weighting models (8)-(10) are correct, and (iv) all four models are incorrect. Details of estimators and specification of the four working models when they are correctly/incorrectly specified are listed in Table 1.

**Figure 2:**
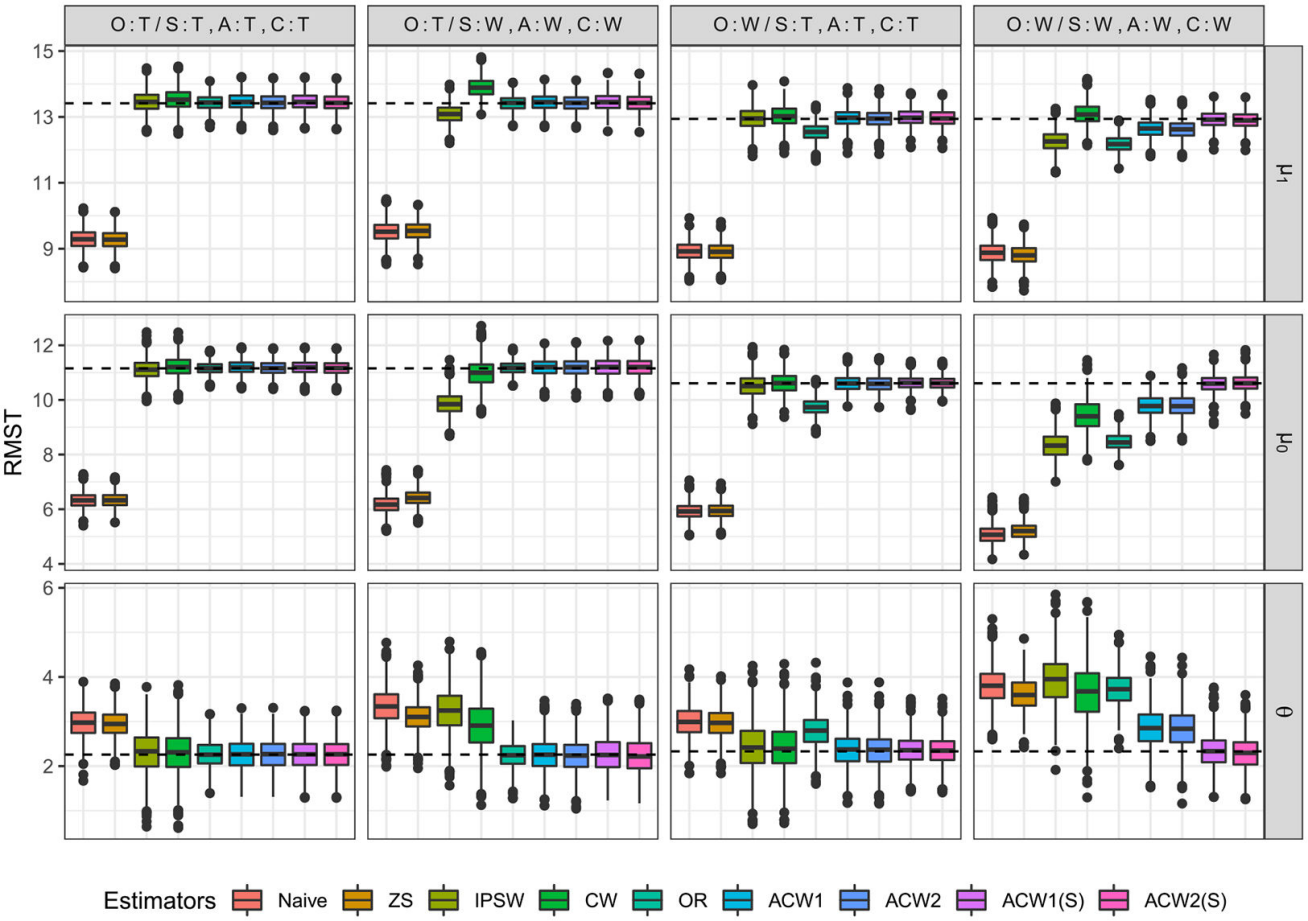
Boxplot of estimators under four model specification scenarios; T: true (correct) model, W: wrong (incorrect) model.

Table 2 and Figure 2 summarize the results based on 1,000 Monte Carlo replications. The bootstrap variance estimation was used for all estimators with *B* = 100. When all models are correctly specified, the Naive and ZS estimators that are based only on the RCT sample show biased estimations of the ATE due to the selection bias in the RCT sample. The OR, IPSW, CW, and ACW estimators correct that bias by leveraging the observational sample covariates. The ACW estimators were found to be more efficient than other unbiased IPW estimators. Even though the variance of the OR estimator is smaller, it is biased when the outcome model is incorrectly specified. The CW and IPSW estimators are biased when the sampling score model is not correctly specified. The proposed ACW estimators are double robust when either the outcome model or the other three models are correctly specified. In Scenario 4 where both the outcome model and the weighting models are misspecified, the ACW estimator using the penalized sieve estimation, i.e., ACW1(S) and ACW2(S), is still unbiased. In Scenario 1 and 2, the efficiencies of the ACW1(S) and ACW2(S) estimators are comparable to that of the ACW1 and ACW2 estimators. In Scenario 3 and 4 where the outcome model is incorrect, ACW1(S) and ACW2(S) are more efficient than their counterparts, confirming that using the method of sieves can improve efficiency. In addition, the ACW2 and ACW2(S) estimators were found to be more consistent than the ACW1 and ACW1(S) estimators in a finite sample.

## 6 Real data application

We apply the proposed method to estimate the effect of adjuvant chemotherapy on survival in patients with early-stage resected non-small cell lung cancer (NSCLC). Cancer and Leukemia Group B (CALGB) 9633 is the only randomized phase III trial designed to evaluate the effectiveness of adjuvant chemotherapy over observation for stage IB NSCLC [40]. An additional observational sample for stage IB NSCLC patients was extracted from National Cancer Database (NCDB), including more than 15,000 patients with the same eligibility criteria as CALGB 9633. More details of the two data sources are given in the study by Lee et al. [5], where they conducted an integrative analysis of the CALGB trial sample and the NCDB sample to improve generalizability for the CALGB 9633 trial-based estimator of average risk of cancer recurrence.

Table 3 summarizes the distribution of the four baseline covariates by the data sources, which have been considered important prognostic factors. The baseline covariates of the patients in the CALGB trial are different from those of the patients in NCDB. Specifically, the CALGB trial patients consist of more males, are younger, and have smaller tumor sizes. Consequently, an important clinical question is whether adjuvant chemotherapy benefits the general stage IB NSCLC patients population, represented by the NCDB, which is a population-based registry capturing approximately 79% of newly diagnosed lung cancers in the United States, and contains more females, are order, and have larger tumor sizes than the CALGB patients. Given that the RCT sample is relatively healthier than the NCDB sample, the estimators based only on CALGB 9633 sample would result in biased estimation of the true effect of adjuvant chemotherapy on the real-world population of early-stage NSCLC patients.

We estimate a 12-year difference of the restricted mean lifetime between adjuvant chemotherapy and observation (i.e., no chemotherapy). The nonparametric bootstrap method is used to estimate the standard errors. The results are given in Table 4, using the proposed estimators and other existing methods in the simulation studies. The Naive and ZS estimators indicate that in the RCT sample, there is no 12-year RMST difference between the adjuvant chemotherapy and observation, i.e., 0.02 and 0.04 years, respectively. All other estimators that utilize the covariate information of the OS show a much larger difference in the RMST. The IPSW and CW estimators show about 0.4–0.5 year increase in RMST for adjuvant chemotherapy over observation, and the OR estimator shows about 0.84 year increase; however, these estimates are not significant. The proposed ACW estimators give an estimate of about a 1-year RMST increase for patients who received adjuvant chemotherapy, which is significant at 0.05 level.

**Figure 3:**
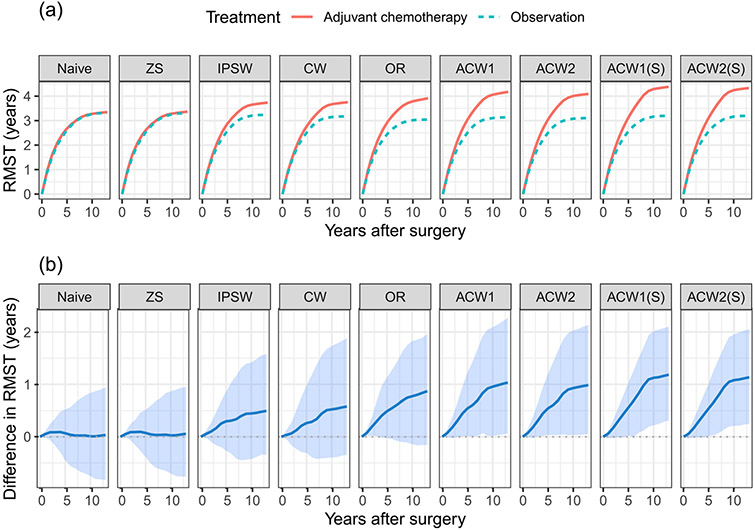
Estimated RMST plots of adjuvant chemotherapy and observation and their difference as a function of restricted times.

Figure 3 represents the estimated restricted mean lifetime for adjuvant chemotherapy and observation and their difference as a function of restricted times *τ*. All selection-bias-adjusted estimators, i.e., the IPSW, CW, OR, ACW1, ACW2, ACW1(S), and ACW2(S) estimators, show a trend of increasing RMST difference over *τ* for all *τ* from 1 to 13 years. Especially, all of the estimators show significant non-zero differences when *τ* is large. Compared to the ACW1 and ACW2 estimators, the ACW1(S) and ACW2(S) estimators gain efficiency by using the method of sieves. On the other hand, the Naive and ZS estimators that are based only on the RCT sample show nearly flat trends near zero difference over *τ*. All these results indicate that the proposed estimators are able to detect the effect of adjuvant chemotherapy on survival in the real world population than that in the RCT sample with higher efficiency and more protection against model misspecification, by leveraging the information from the OS.

This substantial heterogeneity in the treatment effect is mainly due to age and tumor size. While many baseline covariates are prognostic factors for survival risk, age, and tumor size are the few variables associated with the outcome and significantly interact with the treatments. It is the reason that these two variables are of interest for evaluating treatment effect heterogeneity. Subgroup analysis based only on the RCT data supports the same extent of treatment effect heterogeneity varied by age and tumor size. The patients with older age and larger tumor size have significantly less risk for death after adjuvant chemotherapy than those who were younger and had smaller tumors [40]. The trend is consistent with the treatment effect heterogeneity found in the integrated analysis for the target population. The remaining difference in treatment heterogeneity could be caused by the imbalance of the covariates distribution between the RCT sample and the target population, and again the difference is expected and reasonable, and we believe that it indeed reflects the value of such integrated analysis.

## 7 Discussion

This article considers a framework to estimate the treatment effect defined as a function of the treatment-specific survival probability function. The proposed ACW estimators are motivated by two identification formulas based on the survival outcome model and the weighting models and achieve local efficiency and double robustness based on semiparametric theory.

The proposed ACW estimators are similar to the estimator proposed by Zhang and Schaubel [18] that combines the treatment-specific Cox model by Chen and Tsiatis [10] and the IPTW Nelson–Aalen method proposed by Wei [41]. However, unlike our estimator that is derived from the EIF, their approach is based on augmenting the IPW estimating equation to achieve double robustness, and the efficiency of their estimator has not been studied yet. Moreover, their estimator is based only on the RCT sample, whereas we leverage the observational sample to account for selection bias, resulting in an additional augmentation in terms of the sampling score model. In addition, we focus on the class of estimands defined as functionals of the survival functions, including the framework proposed by Zhang and Schaubel [18] as a special case. The proposed approach is also similar to Zhang et al. [19], focusing on the broad class of Mann–Whitney-type causal effect based on the semiparametric theory. To derive the EIF, they construct a RAL estimator that excludes censored data. In contrast, by restricting our interest to the functional of the treatment-specific survival curves, we consider a different RAL estimator utilizing both observed and censored data. The proposed class of estimators could be more robust under highly censored data and covers a broad class of estimands that are favored in time-to-event data.

Instead of a penalized nonparametric sieves method, other machine learning methods can be used, such as survival trees [42] or random forests [43] as alternative to the Cox proportional hazard model. A cross-fitting technique can be employed to remove the Donsker’s condition, which is questionable to hold for these methods [44].

The proposed methods assume that the trial participation is ignorable, i.e., all covariates related to the trial participation and survival time are captured. However, some important covariates might not be available in the observational samples as there were not originally collected for the research purpose. Future work could involve a sensitivity analysis assessing the robustness of the proposed framework in the presence of unmeasured covariates in observational studies [e.g., 45-48].

In addition to the estimation of the ATE, a key challenge is to identify subgroups of patients for whom the treatment is more effective. Estimating individualized treatment effects is a key toward precision medicine so that doctors can tailor the treatment for individual patients given their characteristics. Interesting future research would be generalizing individualized treatment effects for survival outcomes combining RCT and observational studies, following Yang et al. [49,50] and Wu and Yang [51].

## Figures and Tables

**Table 1:** Simulation settings: model specification and estimators; expit(*x*) = {1 + exp(−*x*)}^−1^. O: survival outcome, S: sampling score, A: treatment propensity score, C: censoring

Models		Correctly specified	Incorrectly specified
O	*λ*_1_(*t*∣*X*)	*t* exp(−3.7) exp(−*X*_1_ − *X*_2_ − 1.5*X*_3_)	*t* exp(−0.8) exp(−*e*^*x*_1_^ − *e*^*x*_2_^ − 1.5*X*_3_)
	*λ*_0_(*t*∣*X*)	*t* exp(−3) exp(−1.8*X*_1_ − 1.5*X*_2_ − *X*_3_)	*t* exp(1.5) exp(−1.8*e*^*x*_1_^ − 1.5*e*^*x*_2_^ − *X*_3_)
S	*π*_*δ*_(*X*)	expit{−3.9 − 0.5*X*_1_ − 0.5*X*_2_ − 0.3*X*_3_}	expit{−2.5 − 0.5*e*^*x*_1_^ − 0.5*e*^*x*_2_^ − 0.3*X*_3_}
A	*π*_*A*_	0.5	expit{−1 + 0.5*e*^*x*_1_^ + 0.5*e*^*x*_2_^ − 0.5*e*^*X*_3_^}
	$\lambda_{1}^{C}(t|X)$	*t* exp(−4.5) exp(−0.5*X*_1_ − *X*_2_ − *X*_3_)	*t* exp(−2.5) exp(−0.5*e*^*x*_1_^ − *e*^*x*_2_^ − *X*_3_)
C	$\lambda_{0}^{C}(t|X)$	*t* exp(−3.5) exp(−0.5*X*_1_ − *X*_2_ − *X*_3_)	*t* exp(−1.5) exp(−0.5*e*^*x*_1_^ − *e*^*x*_2_^ − *X*_3_)

Estimators	Details
Naive	${\hat{\theta }}_{\tau }^{\;{}^{\text{Naive}}}=\int_{0}^{\tau }\{{\hat{S}}_{1}^{\;{}^{\text{Naive}}}(t)-{\hat{S}}_{0}^{\;{}^{\text{Naive}}}(t)\}dt$, where ${\hat{S}}_{a}^{\;{}^{\text{Naive}}}(t)=n^{-1}\sum_{i=1}^{N}\delta_{i}A_{ai}\{{\hat{\pi }}_{ai}{\}}^{-1}e^{{\hat{\Lambda }}_{ai}^{C}(t)}Y_{i}(t)$
ZS	The denominator of the estimator proposed by Zhang and Schaubel [18]
OR	${\hat{\theta }}_{\tau }^{\;{}^{\text{OR}}}=\int_{0}^{\tau }\{{\hat{S}}_{1}^{\;{}^{\text{OR}}}(t)-{\hat{S}}_{0}^{\;{}^{\text{OR}}}(t)\}dt$, where ${\hat{S}}_{a}^{\;{}^{\text{OR}}}(t)$ is defined by (4)
IPSW	${\hat{\theta }}_{\tau }^{\;{}^{\text{IPSW}}}=\int_{0}^{\tau }\{{\hat{S}}_{1}^{\;{}^{\text{IPSW}}}(t)-{\hat{S}}_{0}^{\;{}^{\text{IPSW}}}(t)\}dt$ where ${\hat{S}}_{a}^{\;{}^{\text{IPSW}}}(t)=\sum_{i=1}^{N}\delta_{i}{\hat{\omega }}_{i}^{\text{IPSW}}A_{ai}\{{\hat{\pi }}_{ai}{\}}^{-1}e^{{\hat{\Lambda }}_{ai}^{C}(t)}Y_{i}(t)$
CW	${\hat{\theta }}_{\tau }^{\;{}^{\text{CW}}}=\int_{0}^{\tau }\{{\hat{S}}_{1}^{\;{}^{\text{CW}}}(t)-{\hat{S}}_{0}^{\;{}^{\text{CW}}}(t)\}dt$ where ${\hat{S}}_{a}^{\;{}^{\text{CW}}}(t)$ is defined by (11)
ACW1	${\hat{\theta }}_{\tau }^{\;{}^{\text{ACW}1}}=\int_{0}^{\tau }\{{\hat{S}}_{1}^{\;{}^{\text{ACW}1}}(t)-{\hat{S}}_{0}^{\;{}^{\text{ACW}1}}(t)\}dt$ where ${\hat{S}}_{a}^{\;{}^{\text{ACW}1}}(t)$ is defined by (15)
ACW2	${\hat{\theta }}_{\tau }^{\;{}^{\text{ACW}2}}=\int_{0}^{\tau }\{{\hat{S}}_{1}^{\;{}^{\text{ACW}2}}(t)-{\hat{S}}_{0}^{\;{}^{\text{ACW}2}}(t)\}dt$ where ${\hat{S}}_{a}^{\;{}^{\text{ACW}2}}(t)$ is defined by (16)
ACW1(S)	The penalized ACWl estimator using the method of sieves with *g*(*X*) = *g*_2_(*X*)
ACW2(S)	The penalized ACW2 estimator using the method of sieves with *g*(*X*) = *g*_2_(*X*)

**Table 2:** Simulation results under four scenarios; T: true (correct) model, W: wrong (incorrect) model. Bias is the empirical bias of point estimates; ESE is the empirical standard error of estimates; RSE is the relative bias (%) of bootstrap standard error estimates; CP is the empirical coverage probability of the 95% confidence intervals

	BIAS			ESE			RSE (%)			CP (%)		
Estimator	*μ*_1_	*μ*_0_	*θ*	*μ*_1_	*μ*_0_	*θ*	*μ*_1_	*μ*_0_	*θ*	*μ*_1_	*μ*_0_	*θ*
	**Scenario 1: O:T / S:T, A:T, C:T**											
Naive	−4.12	−4.83	0.71	0.30	0.28	0.33	7.44	3.91	4.97	0.0	0.0	39.0
ZS	−4.14	−4.83	0.69	0.29	0.26	0.30	7.12	2.13	4.65	0.0	0.0	34.2
IPSW	0.04	−0.04	0.08	0.32	0.38	0.49	2.33	−0.14	2.01	93.9	94.7	93.3
CW	0.11	0.06	0.05	0.32	0.37	0.50	2.57	1.57	2.29	92.4	93.9	93.7
OR	0.02	0.01	0.01	0.22	0.23	0.30	1.45	−2.59	1.48	94.7	95.3	95.0
ACW1	0.05	0.04	0.00	0.25	0.26	0.35	2.14	−1.60	1.54	93.4	93.9	94.4
ACW2	0.02	0.02	0.00	0.25	0.26	0.35	2.27	−0.31	2.56	94.0	94.5	94.4
ACW1(S)	0.05	0.04	0.00	0.25	0.26	0.34	1.17	−2.46	−0.19	93.4	93.7	94.4
ACW2(S)	0.02	0.02	0.00	0.25	0.26	0.35	1.76	−0.98	1.42	93.9	94.8	94.1
	**Scenario 2: O:T / S:W, A:W, C:W**											
Naive	−3.90	−4.98	1.08	0.31	0.32	0.39	−0.48	2.62	1.88	0.0	0.0	19.5
ZS	−3.88	−4.73	0.85	0.28	0.29	0.33	0.35	1.41	−0.96	0.0	0.0	26.9
IPSW	−0.32	−1.31	0.98	0.28	0.42	0.49	−4.73	0.34	−2.40	82.0	13.2	48.6
CW	0.48	−0.17	0.65	0.30	0.50	0.57	−0.41	−0.92	−2.28	63.6	92.2	79.7
OR	0.01	0.01	−0.01	0.22	0.23	0.28	0.22	−3.62	−3.81	95.2	95.4	96.6
ACW1	0.03	0.03	0.00	0.24	0.31	0.38	−0.30	−7.07	−5.96	94.3	95.4	95.4
ACW2	0.01	0.03	−0.02	0.24	0.32	0.38	−0.23	−2.23	−2.49	94.4	95.0	95.5
ACW1(S)	0.04	0.03	0.01	0.26	0.35	0.42	0.93	−7.55	−7.20	93.0	94.3	94.6
ACW2(S)	0.01	0.03	−0.02	0.26	0.36	0.42	1.27	−1.51	−2.70	93.1	93.6	93.9
	**Scenario 3: O:W / S:T, A:T, C:T**											
Naive	−4.02	−4.68	0.67	0.30	0.29	0.36	4.14	4.17	4.66	0.0	0.0	49.7
ZS	−4.03	−4.66	0.63	0.29	0.28	0.34	3.23	3.77	4.88	0.0	0.0	49.6
IPSW	0.01	−0.09	0.10	0.33	0.41	0.54	1.42	2.55	3.43	94.4	93.6	93.0
CW	0.08	0.00	0.08	0.33	0.40	0.54	1.50	3.73	3.36	93.7	93.1	93.6
OR	−0.40	−0.86	0.47	0.25	0.29	0.37	−2.11	3.94	4.18	64.7	15.2	74.1
ACW1	0.03	0.00	0.03	0.26	0.29	0.37	−0.58	−2.68	−1.27	94.3	93.8	94.7
ACW2	0.01	−0.01	0.02	0.26	0.29	0.37	−0.46	1.54	1.67	94.5	93.8	94.5
ACW1(S)	0.04	0.02	0.02	0.24	0.25	0.32	−4.10	−11.19	−8.99	94.7	94.8	95.0
ACW2(S)	0.02	0.01	0.01	0.24	0.25	0.32	−2.19	−1.54	−1.50	95.1	93.9	94.2
	**Scenario 4: O:W / S:W, A:W, C:W**											
Naive	−4.06	−5.53	1.48	0.34	0.33	0.43	2.18	5.94	3.48	0.0	0.0	7.4
ZS	−4.12	−5.40	1.28	0.31	0.30	0.38	2.67	6.78	4.90	0.0	0.0	8.0
IPSW	−0.68	−2.27	1.60	0.32	0.48	0.55	−2.43	2.19	−2.03	44.1	1.2	20.2
CW	0.14	−1.18	1.33	0.32	0.56	0.65	0.59	1.51	−1.80	92.0	43.0	46.3
OR	−0.76	−2.15	1.39	0.25	0.31	0.38	2.04	0.54	2.45	11.7	0.0	4.9
ACW1	−0.29	−0.82	0.53	0.26	0.39	0.45	2.91	−1.69	−2.36	79.4	43.4	77.1
ACW2	−0.32	−0.82	0.50	0.26	0.40	0.46	2.98	1.53	0.00	75.9	44.2	77.9
ACW1(S)	−0.02	−0.02	0.01	0.24	0.32	0.38	−0.42	−13.16	−11.20	95.1	96.1	95.4
ACW2(S)	−0.04	0.00	−0.04	0.24	0.32	0.38	−0.10	−3.96	−4.13	94.9	96.0	95.3

**Table 3:** Baseline characteristics of the CALGB 9633 trial sample and the NCDB sample; mean (standard deviation) for continuous and number (proportion) for binary covariate

	Male (*X*_1_)	Age (*X*_2_)	Squamous histology (*X*_3_)	Tumor size (*X*_4_)
RCT: CALGB 9633 (*n* = 319)	204 (64%)	60.83 (9.62)	128 (40%)	4.6 (2.08)
OS: NCDB (*n* = 15,379)	8,458 (55%)	67.87 (10.18)	5,998 (39%)	4.94 (3.04)

**Table 4:** Estimates and 95% confidence intervals of 12-year difference of the restricted mean lifetime between adjuvant chemotherapy and observation

Estimator	${\hat{\mu }}_{1}$	${\hat{\mu }}_{0}$	$\hat{\theta }$
Naive	3.33 (2.86, 3.94)	3.31 (2.81, 3.92)	0.022 (−0.815, 0.895)
ZS	3.34 (2.89, 3.94)	3.30 (2.79, 3.90)	0.044 (−0.794, 0.881)
IPSW	3.71 (3.09, 4.55)	3.23 (2.62, 3.83)	0.478 (−0.339, 1.550)
CW	3.73 (3.07, 4.69)	3.17 (2.49, 3.87)	0.561 (−0.363, 1.830)
OR	3.88 (3.17, 4.69)	3.04 (2.37, 3.61)	0.835 (−0.160, 1.890)
ACW1	4.14 (3.42, 5.17)	3.13 (2.45, 3.64)	1.01 (0.048, 2.200)
ACW2	4.07 (3.35, 5.00)	3.10 (2.41, 3.64)	0.967 (0.043, 2.100)
ACW1(S)	4.36 (3.56, 5.17)	3.19 (2.66, 3.69)	1.160 (0.290, 2.087)
ACW2(S)	4.31 (3.50, 5.15)	3.19 (2.66, 3.69)	1.114 (0.239, 2.056)

## Data Availability

The data that support the findings of the paper were assembled through agreements with third parties for licensed use. Without the permission of these third parties and to avoid unintended leakage of patient privacy, we elect to not share the data.

## Appendix

### A EIFs for survival estimands

All survival estimands mentioned in the main paper have the EIF in the form of a combination of weighted integrals of the EIF for treatment-specific survival curves [17]. Specifically, the EIF for *θ*_*τ*_ is

$$\varphi_{\theta_{\tau }}^{\;\text{eff}}(𝒪)=\overset{\;\tau }{\underset{0\;}{\int }}\phi_{1}(t)\varphi_{1}^{\text{eff}}(t;\;𝒪)dt+\overset{\;\tau }{\underset{0\;}{\int }}\phi_{0}(t)\varphi_{0}^{\text{eff}}(t;\;𝒪)dt,$$

where $\varphi_{a}^{\;\text{eff}}(t;\;𝒪)$ is the EIF for treatment-specific survival curve *S*_*a*_(*t*), and *ϕ*_*a*_(·) is a function that $E\{\phi_{a}(\cdot {)}^{2}\}<\infty$, for *a* ∈ {0, 1}.

1. Difference in the survival at a time point $\tau :{\hat{\theta }}_{\tau }={\hat{S}}_{1}(\tau )-{\hat{S}}_{0}(\tau )\;\Rightarrow \;\phi_{1}(t)=\delta (t-\tau )$ and *ϕ*_0_(*t*) = *δ*(*t* + *τ*), where *δ*(·) is the Dirac delta function.
2. Difference in RMSTs up to $\tau :{\hat{\theta }}_{\tau }=\int_{0}^{\tau }{\hat{S}}_{1}(\tau )dt-\int_{0}^{\tau }{\hat{S}}_{0}(\tau )dt\;\Rightarrow \;\phi_{1}(t)=1$ and *ϕ*_0_(*t*) = −1.
3. Ratio of RMTLs up to $\tau :{\hat{\theta }}_{\tau }=\left\{\tau -\int_{0}^{\tau }{\hat{S}}_{1}(\tau )dt\right\}\;∕\;\left\{\tau -\int_{0}^{\tau }{\hat{S}}_{0}(\tau )dt\right\}$. $\Rightarrow \phi_{1}(t)=-{\left\{\tau -\int_{0}^{\tau }{\hat{S}}_{0}(\tau )dt\right\}}^{-1}$ and $\phi_{0}(t)=-{\hat{\theta }}_{\tau }{\left\{\tau -\int_{0}^{\tau }{\hat{S}}_{0}(\tau )dt\right\}}^{-1}$ by the Taylor expansion.
4. Difference in *τ*th quantile of survivals: *θ* = *q*_1,*τ*_ − *q*_0,*τ*_ and $\hat{\theta }={\hat{q}}_{1,\tau }-{\hat{q}}_{0,\tau }$, where *q*_*a,τ*_ = inf_*q*_{*S*_*a*_(*q*) ≤ *τ*} and ${\hat{q}}_{a,\tau }={\text{inf}}_{q}\{{\hat{S}}_{a}(q)\leq \tau \}$.

Following the Bahadur-type representation, under regularity conditions [52], ${\hat{q}}_{a,\tau }$ can be expressed as follows:

$${\hat{q}}_{a,\tau }-q_{a,\tau }=\frac{{\hat{S}}_{a}(q_{a,\tau })-S_{a}(q_{a,\tau })}{{\dot{S}}_{a}(q_{a,\tau })}+o_{p}(N^{-1∕2}),$$

where ${\dot{S}}_{a}(q_{a,\tau })=dS_{a}(q)\;∕\;dq$.

$$\Rightarrow \phi_{1}(t)=\{{\dot{S}}_{1}(q_{1,\tau }){\}}^{-1}I(t=q_{1,\tau })\;\text{and}\;\phi_{0}(t)=\{{\dot{S}}_{0}(q_{0,\tau }){\}}^{-1}I(t=q_{0,\tau }).$$

### B Proofs

#### B.1 Proof of Theorem 1

We first derive the EIF for treatment-specific survival curves without censoring, i.e., under full data set $𝒱=(X,A,T,\delta ,\tilde{\delta })$, and then derive it in the presence of censoring, i.e., under the observed data set $𝒪=(X,A,U,\Delta ,\delta ,\tilde{\delta })$. The proof based on 𝒱 is similar to the one in given in the study by Lee et al. [5] using the method of parametric submodel [53]. We use *ξ* in the subscript to denote the submodel. For example, $f_{\xi }(𝒱)$ is a one-dimensional parametric submodel with the true f(𝒱) at *ξ* = 0, i.e., $f_{\xi }(𝒱){|}_{\xi =0}=f(𝒱)$.

Since the likelihood of a single 𝒱 is

$$f(𝒱)=\{f(X)f(\delta =1|X)f(A|X,\delta =1)f(T|X,A,\delta =1){\}}^{\delta }\{f(X){\}}^{l\tilde{t}a}$$

and $\delta l\tilde{t}a=0$, the score function can be decomposed as follows:

$$\dot{S}(𝒱)=\delta \dot{S}(X)+\delta \dot{S}(\delta |X)+\delta \dot{S}(A|X,\delta =1)+\delta \dot{S}(T|X,A,\delta =1)+\tilde{\delta }\dot{S}(X).$$

We use $\dot{S}$ to represent the score function.

The nuisance tangent space from the semiparametric theory is

$$H=H_{1}⊕H_{2}⊕H_{3}⊕H_{4},$$

where

$$H_{1}=\{h(X)\;:E\{h(X)\}=0\}\;\;H_{2}=\{h(X,\delta )\;:E\{h(\delta ,X)|X\}=0\}\;\;H_{3}=\{h(A,\delta ,X)\;:E\{h(A,\delta ,X)|\delta ,X\}=0\}\;\;H_{4}=\{h(T,A,\delta ,X)\;:E\{h(T,A,\delta ,X)|A,\delta ,X\}=0\}.$$

Here, we only derive the EIF for *S*_1_(*u*). The EIF for *S*_0_(*u*) can be easily derived using the similar technique. We denote the EIF for *S*_1_(*u*) under 𝒱 as $\varphi_{1}^{F}(u|𝒱)$, which must satisfy $\varphi_{1}^{F}(u|𝒱)\in H$ and ∣∂∂ξS1,ξ(u)∣ξ=0=E{φ1F(u∣𝒱)S.(𝒱)}, where $S_{1,\xi }(u)=E\{\tilde{\delta }dS_{1,\xi }(u,X)\}$. Toward this end, we express

(B1)
∣∂∂ξS1,ξ(u)∣ξ=0=∣∫δ~dS1,ξ(u,X)fξ(𝒱)d𝒱∣ξ=0=∣∫δ~dS1,ξ(u,X)S.ξ(V)fξ(𝒱)d𝒱∣ξ=0+∣∫δ~d{∂∂ξS1,ξ(u,X)}fξ(𝒱)d𝒱∣ξ=0=E{δ~dS1(u,X)S.ξ(X)}+E{∂∂ξS1,ξ(u,X)∣ξ=0}.

For the first term in (B1), we have

(B2)
$$E\{\tilde{\delta }dS_{1}(u,X)\dot{S}(X)\}=E[l\tilde{t}ad\{S_{1}(u,X)-S_{1}(u)\}]\;\;=E[l\tilde{t}ad\{S_{1}(u,X)-S_{1}(u)\}\{\dot{S}(X,A,T,\delta )+l\tilde{t}a\dot{S}(X)\}]\;\;=E[l\tilde{t}ad\{S_{1}(u,X)-S_{1}(u)\}\dot{S}(𝒱)],$$

and for the second term in (B1), we have

(B3)
∣∂∂ξS1,ξ(u∣X)∣ξ=0=∣∫I(T≥u)∂∂ξfξ(u∣X,δ=1,A=1)du∣ξ=0=∣∫I(T≥u)S.ξ(T∣X,δ=1,A=1)fξ(u∣X,δ=1,A=1)du∣ξ=0=∫I(T≥u)S.(T∣X,δ,A)δπδ(X)AπA(X)f(t∣X)du=E{δπδ(X)AπA(X)I(T≥u)S.(T∣X,δ,A)∣X)}.

By combining (B2) and (B3),

E{{∂∂ξS1,ξ(u,X)∣ξ=0}=E{δπδ(X)AπA(X)I(T≥u)S.(T∣X,δ,A)}=E[δπδ(X)AπA(X){I(T≥u)−S1(u,X)}S.(T∣X,δ,A)]=E[δπδ(X)AπA(X){I(T≥u)−S1(u,X)}S.(T,X,δ,A)]=E[δπδ(X)AπA(X){I(T≥u)−S1(u,X)}S.(𝒱)].

Therefore,

∣∂∂ξS1,ξ(u)∣ξ=0=E([δπδ(X)AπA(X){I(T≥u)−S1(u,X)}+δ~d{S1(u,X)−S1(u)}]S.(𝒱)).

Let

$$\varphi_{1}^{F}(u|𝒱)=\frac{\delta }{\pi_{\delta }(X)}\frac{A}{\pi_{A}(X)}\{I(T\geq u)-S_{1}(u,X)\}+\tilde{\delta }dS_{1}(u,X)-S_{1}(u).$$

Since

$$\left\{\tilde{\delta }d-\frac{\delta }{\pi_{\delta }(X)}\frac{A}{\pi_{A}(X)}\right\}S_{1}(u,X)\in H_{3}$$

and

$$\frac{\delta }{\pi_{\delta }(X)}\frac{A}{\pi_{A}(X)}I(T\geq u)-S_{1}(u)\in H_{4},$$

we have $\varphi_{1}^{F}(u|𝒱)\in H$; thus, $\varphi_{1}^{F}(u|𝒱)$ is the EIF for *S*_1_(*u*).

Now, in the presence of censoring, we observe data set $𝒪=(X,A,U,\Delta ,\delta ,\tilde{\delta })$ instead of 𝒱. Following Robins et al. [54] and Tsiatis [30], the EIF for the monotone coarsened data is given as follows:

$$\frac{\delta }{\pi_{\delta }(X)}\frac{A}{\pi_{A}(X)}\frac{\Delta }{S_{1}^{C}(U,X)}I(U\geq u)-S_{1}(u)-\frac{\delta }{\pi_{\delta }(X)}\frac{A}{\pi_{A}(X)}\{S_{1}(u,X)-\tilde{\delta }d\}S_{1}(u,X)\;\;+\int \frac{\delta }{\pi_{\delta }(X)}\frac{A}{\pi_{A}(X)}\frac{dM_{1}^{C}(r,X)}{S_{1}^{C}(r,X)}g(u,r,X),$$

where $S_{1}^{C}(r,X)=p(C\geq r|X,A=1,\delta =1)$ and $dM_{1}^{C}(r,X)=dN^{C}(r)-\lambda_{1}^{C}(r,X)Y(r),\;N^{C}(r)=I(U\leq r,\Delta =1)$ and *Y*(*r*) = *I*(*U* ≥ *r*). According to Theorem 10.4 from Tsiatis [30], the optimal *g*(*u*, *r*, *X*) is

g(u,r,X)=E{I(T≥u∣X,A=1,δ=1,T≥r)}.

Thus,

(B4)
$$\int \frac{\delta }{\pi_{\delta }(X)}\frac{A}{\pi_{A}(X)}\frac{dM_{1}^{C}(r,X)}{S_{1}^{C}(r,X)}g(u,r,X)\;\;=\overset{\;\infty }{\underset{0\;}{\int }}\frac{\delta }{\pi_{\delta }(X)}\frac{A}{\pi_{A}(X)}\frac{dM_{1}^{C}(r,X)}{S_{1}^{C}(r,X)}\left\{I(u>r)\frac{S_{1}(u,X)}{S_{1}(r,X)}+I(r\geq u)\cdot \;1\right\}=\overset{\;u}{\underset{0\;}{\int }}\frac{\delta }{\pi_{\delta }(X)}\frac{A}{\pi_{A}(X)}\frac{dM_{1}^{C}(r,X)}{S_{1}^{C}(r,X)}\frac{S_{1}(u,X)}{S_{1}(r,X)}+\overset{\;\infty }{\underset{u\;}{\int }}\frac{\delta }{\pi_{\delta }(X)}\frac{A}{\pi_{A}(X)}\frac{dM_{1}^{C}(r,X)}{S_{1}^{C}(r,X)}.$$

For the second term in (B4), we can express as follows:

(B5)
$$(B4)=\frac{\delta }{\pi_{\delta }(X)}\frac{A}{\pi_{A}(X)}\left\{\overset{\;\infty }{\underset{u\;}{\int }}\frac{dN^{C}(r)}{S_{1}^{C}(r,X)}-\overset{\;\infty }{\underset{u\;}{\int }}\frac{\lambda_{1}(r,X)Y(r)}{S_{1}^{C}(r,X)}\right\}\;\;=\frac{\delta }{\pi_{\delta }(X)}\frac{A}{\pi_{A}(X)}Y(u)\left\{\frac{1-\Delta }{S_{1}^{C}(U,X)}-\overset{\;U}{\underset{u\;}{\int }}\frac{\lambda_{1}(r,X)}{S_{1}^{C}(r,X)}\right\}\;\;=\frac{\delta }{\pi_{\delta }(X)}\frac{A}{\pi_{A}(X)}Y(u)\left[\frac{1-\Delta }{S_{1}^{C}(U,X)}-\left\{\frac{1}{S_{1}^{C}(U,X)}-\frac{1}{S_{1}^{C}(u,X)}\right\}\right]\;\;=\frac{\delta }{\pi_{\delta }(X)}\frac{A}{\pi_{A}(X)}Y(u)\left\{\frac{1}{S_{1}^{C}(u,X)}-\frac{\Delta }{S_{1}^{C}(U,X)}\right\}.$$

Plugging (B5) to (B4), the EIF for *S*_1_(*u*) under 𝒪 is

$$\frac{\delta }{\pi_{\delta }(X)}\frac{A}{\pi_{A}(X)}\frac{\Delta }{S_{1}^{C}(U,X)}I(U\geq u)-S_{1}(u)-\frac{\delta }{\pi_{\delta }(X)}\frac{A}{\pi_{A}(X)}\{S_{1}(u,X)-\tilde{\delta }d\}S_{1}(u,X)\;\;+\overset{\;u}{\underset{0\;}{\int }}\frac{\delta }{\pi_{\delta }(X)}\frac{A}{\pi_{A}(X)}\frac{dM_{1}^{C}(r,X)}{S_{1}^{C}(r,X)}\frac{S_{1}(u,X)}{S_{1}(r,X)}-\frac{\delta }{\pi_{\delta }(X)}\frac{A}{\pi_{A}(X)}Y(u)\left\{\frac{1}{S_{1}^{C}(u,X)}-\frac{\Delta }{S_{1}^{C}(U,X)}\right\}\;\;=\frac{\delta }{\pi_{\delta }(X)}\frac{A}{\pi_{A}(X)}\frac{I(U\geq u)}{S_{1}^{C}(u,X)}-S_{1}(u)-\frac{\delta }{\pi_{\delta }(X)}\frac{A}{\pi_{A}(X)}\{S_{1}(u,X)-\tilde{\delta }d\}S_{1}(u,X)\;\;+\overset{\;u}{\underset{0\;}{\int }}\frac{\delta }{\pi_{\delta }(X)}\frac{A}{\pi_{A}(X)}\frac{dM_{1}^{C}(r,X)}{S_{1}^{C}(r,X)}\frac{S_{1}(u,X)}{S_{1}(r,X)}.$$

The EIF for *S*_0_(*t*) can be obtained by analogy.

#### B.2 The influence function of the ACW estimators

For both ACW1 and ACW2 estimators, by the definition of the EIF,

$$\sqrt{N}\{{\hat{S}}_{a}^{{}^{\text{ACW}}}(t)-S_{a}(t)\}=\frac{1}{\sqrt{N}}\sum_{i=1}^{N}\varphi_{a}^{\text{eff}}(t;\;{𝒪}_{i})+o_{p}(1),\;\;\text{for}\;a\in \{0,1\}.$$

Thus, under asymptotic linear characterization of the ATE estimator ${\hat{\theta }}_{\tau }^{\;{}^{\text{ACW}}}=\Psi_{\tau }({\hat{S}}_{1}^{\;{}^{\text{ACW}}}(t),{\hat{S}}_{0}^{\;{}^{\text{ACW}}}(t))$,

$$\sqrt{N}({\hat{\theta }}_{\tau }^{\;{}^{\text{ACW}}}-\theta_{\tau })=\overset{\;\tau }{\underset{0\;}{\int }}\phi_{1}(t)\sqrt{N}\{{\hat{S}}_{1}^{\;{}^{\text{ACW}}}(t)-S_{1}(t)\}dt+\overset{\;\tau }{\underset{0\;}{\int }}\phi_{0}(t)\sqrt{N}\{{\hat{S}}_{0}^{\;{}^{\text{ACW}}}(t)-S_{0}(t)\}dt+o_{p}(1)\;\;=\overset{\;\tau }{\underset{0\;}{\int }}\phi_{1}(t)\frac{1}{\sqrt{N}}\sum_{i=1}^{N}\varphi_{1}^{\text{eff}}(t;\;{𝒪}_{i})dt+\overset{\;\tau }{\underset{0\;}{\int }}\phi_{0}(t)\frac{1}{\sqrt{N}}\sum_{i=1}^{N}\varphi_{0}^{\text{eff}}(t;\;{𝒪}_{i})dt+o_{p}(1)\;\;=\frac{1}{\sqrt{N}}\sum_{i=1}^{N}\left\{\overset{\;\tau }{\underset{0\;}{\int }}\phi_{1}(t)\varphi_{1}^{\text{eff}}(t;\;{𝒪}_{i})dt+\overset{\;\tau }{\underset{0\;}{\int }}\phi_{0}(t)\varphi_{0}^{\text{eff}}(t;\;{𝒪}_{i})dt\right\}+o_{p}(1).$$

Thus, ${\hat{\theta }}_{\tau }^{\;{}^{\text{ACW}}}$ has the influence function:

$$\varphi_{\theta_{\tau }}^{\;\text{eff}}(𝒪)=\overset{\;\tau }{\underset{0\;}{\int }}\phi_{1}(t)\varphi_{1}^{\;\text{eff}}(t;\;𝒪)dt+\overset{\;\tau }{\underset{0\;}{\int }}\phi_{0}(t)\varphi_{0}^{\;\text{eff}}(t;\;𝒪)dt.$$

#### B.3 Proof of Theorem 2

Standard regularity conditions for the consistency of survival OR parameters and treatment propensity score model [e.g., 18,55] and suitable regularity conditions for the M-estimation theory [e.g., 56] are assumed throughout this proof. The former includes almost sure boundedness of *X* and finite cumulative baseline hazard function for survival and censoring, and the latter includes smoothness and identifiability of working models and applicability of dominated convergence theorem.

Under Assumptions 1-3, we consider three cases: (i) survival OR model (O) in (3) is correctly specified, (ii) the weighting models, i.e., the sampling score model (S) in (8), the treatment propensity score model (A) in (9), and the censoring model (C) in (10), are correctly specified; (iii) all working models in (3) and (8)-(10) are correctly specified. Assume that $\hat{\zeta }=(\hat{\eta },\hat{\rho },{\hat{\beta }}_{1},{\hat{\beta }}_{0},{\hat{\gamma }}_{1},{\hat{\gamma }}_{0})$ converges to some $\zeta^{*}=(\eta^{*},\rho^{*},\beta_{1}^{*},\beta_{0}^{*},\gamma_{1}^{*},\gamma_{0}^{*})$, not necessary true. Then, ${\hat{\pi }}_{\delta }(X)=\pi_{\delta }(X;\;\hat{\eta })=\text{exp}\{{\hat{\eta }}^{T}g(X)\}$ converges to $\pi_{\delta }^{*}(X)$, ${\hat{\pi }}_{A}(X)=\pi_{A}(X;\;\hat{\rho })=[1+\text{exp}\{-{\hat{\rho }}^{T}g(X)\}{]}^{-1}$ converges to $\pi_{A}^{*}(X)$, ${\hat{\Lambda }}_{a}(t)={\hat{\Lambda }}_{a}(t;{\hat{\beta }}_{a})$ converges to $\Lambda_{a}^{*}(t)$, and ${\hat{\Lambda }}_{a}^{C}(t)={\hat{\Lambda }}_{a}^{C}(t;\;{\hat{\gamma }}_{a})$ converges to $\Lambda_{a}^{*C}(t)$, for *a* ∈ {0, 1}. The following proof is similar to the one given in the study by Zhang and Schaubel [18] and Zhang et al. [19].

##### B.3.1 Double robustness

We first demonstrate the double robustness property of ${\hat{S}}_{1}^{\;{}^{\text{ACW}1}}(t)$. It is straightforward to show double robustness of ${\hat{S}}_{0}^{\;{}^{\text{ACW}1}}(t)$ and ${\hat{\theta }}^{\;{}^{\text{ACW}1}}$ by analogy. For the simplicity, we only consider the simple random sampling in the OS, but it can be easily extended to the general setting with known design weights. Define ${𝒮}_{1}(t;\;\zeta^{*})\;\;=\;\;\{\pi_{\delta }^{*}(X)\pi_{A}^{*}(X)e^{-\Lambda_{a}^{*C}(t)}{\}}^{-1}\delta AY(t)-\{\pi_{\delta }^{*}(X)\pi_{A}^{*}(X){\}}^{-1}\delta \{A-\pi_{A}^{*}(X)\}e^{-\Lambda_{1}^{*}(t)}-\{\pi_{\delta }^{*}(X{)}^{-1}\delta -\tilde{\delta }d\}e^{-\Lambda_{1}^{*}(t)}\;\;+\{\pi_{\delta }^{*}(X)\pi_{A}^{*}(X){\}}^{-1}\delta A\int_{0}^{t}\{e^{-\Lambda_{1}^{*C}(t)}e^{-\Lambda_{1}^{*}(u)}{\}}^{-1}e^{-\Lambda_{1}^{*}(t)}dM_{1}^{*C}(u)$. Under mild regularity conditions, ${\hat{S}}_{1}^{\;{}^{\text{ACW}1}}(t)\overset{\;p\;}{\to }E\{{𝒮}_{1}(t;\;\zeta^{*})\}$, where

(B6)
$$E\{{𝒮}_{1}(t;\;\zeta^{*})\}=E\left\{\frac{\delta }{\pi_{\delta }^{*}(X)}\frac{A}{\pi_{A}^{*}(X)}\frac{Y(t)}{e^{-\Lambda_{a}^{*C}(t)}}\right\},$$

(B7)
$$E\{{𝒮}_{1}(t;\;\zeta^{*})\}-E\left[\frac{\delta }{\pi_{\delta }^{*}(X)}\left\{\frac{A-\pi_{A}^{*}(X)}{\pi_{A}^{*}(X)}\right\}e^{-\Lambda_{1}^{*}(t)}\right],$$

(B8)
$$E\{{𝒮}_{1}(t;\;\zeta^{*})\}-E\left[\left\{\frac{\delta }{\pi_{\delta }^{*}(X)}-\tilde{\delta }d\right\}e^{-\Lambda_{1}^{*}(t)}\right],$$

(B9)
$$E\{{𝒮}_{1}(t;\;\zeta^{*})\}+E\left\{\frac{\delta }{\pi_{\delta }^{*}(X)}\frac{A}{\pi_{A}^{*}(X)}\overset{\;t}{\underset{0\;}{\int }}\frac{dM_{1}^{*C}(u)}{e^{-\Lambda_{1}^{*C}(t)}}\frac{e^{-\Lambda_{1}^{*}(t)}}{e^{-\Lambda_{1}^{*}(u)}}\right\}.$$

First, consider condition (i) that the model for O is correct. Using the notation *κ*(*t*) from the study by Zhang and Schaubel [18], we can express *Y*(*t*) = *I*(*T* ≥ *t*)*κ*(*t*), where *κ*(*t*) = *I*(*C* ≥ *T* or *C* ≥ *t*). Following Tsiatis [30, Chapter 9.3 and Lemma 10.4], we can write

$$\frac{\kappa (t)}{e^{-\Lambda_{1}^{*C}(t)}}=1-\overset{\;t}{\underset{0\;}{\int }}\frac{dM_{1}^{*C}(u)}{e^{-\Lambda_{1}^{*C}(t)}}.$$

Then, we have

(B10)
$$(B6)=E\left\{\frac{\delta }{\pi_{\delta }^{*}(X)}\frac{A}{\pi_{A}^{*}(X)}\frac{I(T\geq t)\kappa (t)}{e^{-\Lambda_{1}^{*C}(t)}}\right\}=E\left\{\frac{\delta }{\pi_{\delta }^{*}(X)}\frac{A}{\pi_{A}^{*}(X)}I(T\geq t)-\frac{\delta }{\pi_{\delta }^{*}(X)}\frac{A}{\pi_{A}^{*}(X)}\overset{\;t}{\underset{0\;}{\int }}\frac{dM_{1}^{*C}(u)}{e^{-\Lambda_{1}^{*C}(t)}}I(T\geq t)\right\},$$

(B11)
$$(B7)+(B7)=-E\left\{\frac{\delta }{\pi_{\delta }^{*}(X)}\frac{A}{\pi_{A}^{*}(X)}e^{-\Lambda_{1}^{*}(t)}\right\}$$

(B12)
$$+E\{\tilde{\delta }de^{-\Lambda_{1}^{*}(t)}\}.$$

Combining (B9) with (B10)-(B12), we have

(B13)
$$E\{\tilde{\delta }de^{-\Lambda_{1}^{*}(t)}\}$$

(B14)
$$+E\left[\frac{\delta }{\pi_{\delta }^{*}(X)}\frac{A}{\pi_{A}^{*}(X)}\{I(T\geq t)-e^{-\Lambda_{1}^{*}(t)}\}\right]$$

(B15)
$$+E\left[\frac{\delta }{\pi_{\delta }^{*}(X)}\frac{A}{\pi_{A}^{*}(X)}\overset{\;t}{\underset{0\;}{\int }}\frac{dN_{1}^{C}(u)}{e^{-\Lambda_{1}^{*C}(t)}}\left\{I(T\geq t)-\frac{e^{-\Lambda_{1}^{*}(t)}}{e^{-\Lambda_{1}^{*}(u)}}\right\}\right]$$

(B16)
$$-E\left[\frac{\delta }{\pi_{\delta }^{*}(X)}\frac{A}{\pi_{A}^{*}(X)}\overset{\;t}{\underset{0\;}{\int }}\frac{Y(u)d\Lambda_{1}^{*C}(u)}{e^{-\Lambda_{1}^{*C}(t)}}\left\{I(T\geq t)-\frac{e^{-\Lambda_{1}^{*}(t)}}{e^{-\Lambda_{1}^{*}(u)}}\right\}\right].$$

Since $e^{-\Lambda_{1}^{*}(t)}=p(T\geq t|X,A=1,\delta =1)$, (B13) equals to *S*_1_(*t*) and (B14) is 0 by iterated expectation conditioning on {*X*, *A* = 1, *δ* = 1). Also (B15) and (B16) are 0 by iterated expectations conditioning on (*X*, *A* = 1, *δ* = 1, *C* = *u*, *T* ≥ *u*) and (*X*, *A* = 1, *δ* = 1, *C* ≥ *u*, *T* ≥ *u*), respectively.

Now, consider the condition (ii) that the models for S, A, and C are correct. As $\pi_{\delta }^{*}(X)=p(\delta =1|X)$, $\pi_{A}^{*}(X)=p(A=1|X,\delta =1)$, and $e^{-\Lambda_{1}^{*C}(t)}=p(C\geq t|X,A=1,\delta =1)$,

$$(B6)=E\left[\frac{\delta }{\pi_{\delta }^{*}(X)}E\left\{\frac{A}{\pi_{A}^{*}(X)}\frac{I(T\geq t)I(C\geq t)}{e^{-\Lambda_{a}^{*C}(t)}}|X,\delta =1\right\}\right]\;\;=E\left\{\frac{\delta }{\pi_{\delta }^{*}(X)}\frac{\pi_{A}^{*}(X)}{\pi_{A}^{*}(X)}\frac{P(T\geq t|X,A=1,\delta =1)P(C\geq t|X,A=1,\delta =1)}{e^{-\Lambda_{a}^{*C}(t)}}\right\}\;\;=E\{\tilde{\delta }dP(T\geq t|X,A=1,\delta =1)\}\;\;=S_{1}(t)\;\;(B7)+(B8)=E\left[e^{-\Lambda_{1}^{*}(t)}\left\{\tilde{\delta }d-\frac{\delta }{\pi_{\delta }^{*}(X)}\frac{\pi_{A}^{*}(X)}{\pi_{A}^{*}(X)}\right\}\right]=0\;\;(B9)=E\left[\frac{\delta }{\pi_{\delta }^{*}(X)}\frac{\pi_{A}^{*}(X)}{\pi_{A}^{*}(X)}E\left\{\overset{\;t}{\underset{0\;}{\int }}\frac{dM_{1}^{*C}(u)}{e^{-\Lambda_{1}^{*C}(t)}}\frac{e^{-\Lambda_{1}^{*}(t)}}{e^{-\Lambda_{1}^{*}(u)}}|X,A=1,\delta =1\right\}\right]=0.$$

Thus, ${\hat{S}}_{0}^{\;{}^{\text{ACW}1}}(t)$ has the double robustness property. By analogy, it is straightforward to show that ${\hat{S}}_{0}^{\;{}^{\text{ACW}1}}(t)$ converges to $E\{{𝒮}_{0}(t;\zeta^{*})\}$ which equals to *S*_0_(*t*) if either working model for O or weighting models S, A, and C are correct. Consequently, ${\hat{\theta }}_{\tau }^{\;{}^{\text{ACW}1}}$ converges to $E\{\vartheta_{\tau }(\zeta^{*})\}$, where

$$\vartheta_{\tau }(\zeta^{*})=\overset{\;\tau }{\underset{0\;}{\int }}\phi_{1}(t){𝒮}_{1}(t;\;\zeta^{*})dt+\overset{\;\tau }{\underset{0\;}{\int }}\phi_{0}(t){𝒮}_{0}(t;\;\zeta^{*})dt.$$

Then, $E\{\vartheta_{\tau }(\zeta^{*})\}$ equals to *θ*_*τ*_ with the same double robustness properties.

For the ACW2 estimator, we can show that the numerator $-d{\hat{S}}_{1}^{\;{}^{\text{ACW}1}}(u)$ converges in probability to $-dS_{1}(u)$, thus ${\hat{S}}_{1}^{\;{}^{\text{ACW}2}}(t)\overset{\;p\;}{\to }S_{1}(t)$. Similarly, one can easily obtain ${\hat{S}}_{0}^{\;{}^{\text{ACW}2}}(t)\overset{\;p\;}{\to }S_{0}(t)$ and ${\hat{\theta }}_{\tau }^{\;{}^{\text{ACW}2}}\overset{\;p\;}{\to }\theta_{\tau }$.

##### B.3.2 Asymptotic normality

Following empirical process literature, define P as the true measure, $P_{N}$ as the empirical measure, and define $G_{N}=\sqrt{N}(P_{N}-P)$ for the empirical processes. Following the technique used in the study by Zhang et al. [19], we assume that *ζ* and *ζ** take values in a suitable Banach space with

(B17)
$$\sqrt{N}(\hat{\eta }-\eta^{*})=G_{N}\varphi_{\eta }(𝒪)+o_{p}(1)\;\;\sqrt{N}(\hat{\rho }-\rho^{*})=G_{N}\varphi_{\rho }(𝒪)+o_{p}(1)\;\;\sqrt{N}({\hat{\beta }}_{a}-\beta_{a}^{*})=G_{N}\varphi_{\beta_{a}}(𝒪)+o_{p}(1)\;\;\sqrt{N}({\hat{\gamma }}_{a}-\gamma_{a}^{*})=G_{N}\varphi_{\gamma_{a}}(𝒪)+o_{p}(1),$$

for *a* ∈ {0, 1}. Assuming that *ϑ*_*τ*_(*ζ**) belongs to Donsker classes [57,58], and under assumed regularity conditions, $P_{N}\vartheta_{\tau }(\hat{\zeta })={\hat{\theta }}_{\tau }^{\;{}^{\text{ACW}1}}$ and $P\vartheta_{\tau }(\zeta^{*})=\theta_{\tau }$. Then,

(B18)
$$\sqrt{N}\{{\hat{\theta }}_{\tau }^{\;{}^{\text{ACW1}}}-\theta_{\tau }\}=\sqrt{N}\{P_{N}\vartheta_{\tau }(\hat{\zeta })-P\vartheta_{\tau }(\zeta^{*})\}\;\;=G_{N}\vartheta_{\tau }(\hat{\zeta })+\sqrt{N}P\{\vartheta_{\tau }(\hat{\zeta })-\vartheta_{\tau }(\zeta^{*})\}\;\;=G_{N}\vartheta_{\tau }(\zeta^{*})+\sqrt{N}P\{\vartheta_{\tau }(\hat{\zeta })-\vartheta_{\tau }(\zeta^{*})\}+o_{p}(1).$$

As *ϑ* is differentiable at *ζ**, we have the derivative

$$D^{\text{ACW}}=\left(D_{\eta }^{\text{ACW}},D_{\rho }^{\text{ACW}},D_{\beta_{1}}^{\text{ACW}},D_{\beta_{0}}^{\text{ACW}},D_{\gamma_{1}}^{\text{ACW}},D_{\gamma_{0}}^{\text{ACW}}\right)=\overset{\;\tau }{\underset{0\;}{\int }}\{\phi_{1}(t)D{𝒮}_{1}+\phi_{0}(t)D{𝒮}_{0}\}dt.$$

This implies that

$$\sqrt{N}P\{\vartheta_{\tau }(\hat{\zeta })-\vartheta_{\tau }(\zeta^{*})\}=D^{\text{ACW}}\sqrt{N}(\hat{\eta }-\eta^{*},\hat{\rho }-\rho^{*},{\hat{\beta }}_{1}-\beta_{1}^{*},{\hat{\beta }}_{0}-\beta_{0}^{*},{\hat{\gamma }}_{1}-\gamma_{1}^{*},{\hat{\gamma }}_{0}-\gamma_{0}^{*})+o_{p}(1)\;\;=G_{N}\left(D_{\eta }^{\text{ACW}}\varphi_{\eta }+D_{\rho }^{\text{ACW}}\varphi_{\rho }+D_{\beta_{1}}^{\text{ACW}}\varphi_{\beta_{1}}+D_{\beta_{0}}^{\text{ACW}}\varphi_{\beta_{0}}+D_{\gamma_{1}}^{\text{ACW}}\varphi_{\gamma_{1}}+D_{\gamma_{0}}^{\text{ACW}}\varphi_{\gamma_{0}}\right)+o_{p}(1).$$

Therefore,

$$\sqrt{N}\{{\hat{\theta }}_{\tau }^{\;{}^{\text{ACW}1}}-\theta_{\tau }\}=G_{N}\{\vartheta_{\tau }(\zeta^{*})+D_{\eta }^{\text{ACW}}\varphi_{\eta }+D_{\rho }^{\text{ACW}}\varphi_{\rho }+D_{\beta_{1}}^{\text{ACW}}\varphi_{\beta_{1}}+D_{\beta_{0}}^{\text{ACW}}\varphi_{\beta_{0}}+D_{\gamma_{1}}^{\text{ACW}}\varphi_{\gamma_{1}}+D_{\gamma_{0}}^{\text{ACW}}\varphi_{\gamma_{0}}\}+o_{p}(1).$$

The similar technique can be used to prove the asymptotic normality of the ACW2 estimator using the Delta method and the M-estimation theory.

##### B.3.3 Local efficiency

Define the asymptotic variance of the ACW1 estimator as $E(\varsigma_{1}^{2})$, where

$$\varsigma_{1}=\vartheta_{\tau }(\zeta^{*})+D_{\eta }^{\text{ACW}}\varphi_{\eta }+D_{\rho }^{\text{ACW}}\varphi_{\rho }+D_{\beta_{1}}^{\text{ACW}}\varphi_{\beta_{1}}+D_{\beta_{0}}^{\text{ACW}}\varphi_{\beta_{0}}+D_{\gamma_{1}}^{\text{ACW}}\varphi_{\gamma_{1}}+D_{\gamma_{0}}^{\text{ACW}}\varphi_{\gamma_{0}}.$$

When the model for O is correct, then $D_{\beta_{1}}^{\text{ACW}}=0$ and $D_{\beta_{0}}^{\text{ACW}}=0$. When the weighting models S, A, and C are correct, then $D_{\eta }^{\text{ACW}}$, $D_{\rho }^{\text{ACW}}$, $D_{\gamma_{1}}^{\text{ACW}}$, and $D_{\gamma_{0}}^{\text{ACW}}$ are all zero. Thus, when all four working models are correct, then

$$\sqrt{N}\{{\hat{\theta }}_{\tau }^{\;{}^{\text{ACW}1}}-\theta_{\tau }\}=G_{N}\vartheta_{\tau }(\zeta^{*})+o_{p}(1)=G_{N}\varphi_{\theta_{\tau }}^{\;\text{eff}}(𝒪)+o_{p}(1),$$

i.e., $\varsigma_{1}=\varphi_{\theta_{\tau }}^{\;\text{eff}}(𝒪)$, implying that the ACW1 estimator is the locally efficient estimator. The efficiency of the ACW2 estimator can be proved using the similar technique and the small order difference between the ACW1 and the ACW2 estimator.

#### B.4 Proof of Theorem 3

From (B18) in B.3, we want to show that $P\{\vartheta_{\tau }(\hat{\zeta })-\vartheta_{\tau }(\zeta^{*})\}$ is negligible under conditions in Theorem 3. Toward this end, we write

$$P\{\vartheta_{\tau }(\hat{\zeta })-\vartheta_{\tau }(\zeta^{*})\}=\sum_{a=0}^{1}\overset{\;\tau }{\underset{0\;}{\int }}\phi_{a}(t)P\{{𝒮}_{a}(t;\;\hat{\zeta })-{𝒮}_{a}(t;\zeta^{*})\}dt,$$

where ${𝒮}_{a}(t;\;\hat{\zeta })$ is defined in B.3. As *Y*(*t*) = *I*(*T* ≥ *t*)*κ*(*t*) with *κ*(*t*) = *I*(*C* ≥ *T* or *C* ≥ *t*) and $e^{\Lambda_{a}^{*C}(t)}\kappa (t)=1-\int_{0}^{t}e^{\Lambda_{a}^{*C}(u)}dM_{a}^{*C}(u)$, by iterated expectation, we have

(B19)
P{𝒮a(t;ζ^)−𝒮a(t;ζ∗)}=P[δπδ(X;η^)AπA(X;ρ^){Sa(t,X)−Sa(t,X;β^a)}+lt~adSa(t,X;β^a)−Sa(t,X)][−δπδ(X;η^)AπA(X;ρ^)∫0tdM^aC(u;γ^a)SaC(t,X;γ^a){Sa(t,X)−Sa(t,X;β^a)Sa(u,X;β^a)}],

(B20)
$$=P\left[\left\{\frac{\delta }{\pi_{\delta }(X;\;\hat{\eta })}\frac{A}{\pi_{A}(X;\;\hat{\rho })}-1\right\}\{S_{a}(t,X)-S_{a}(t,X;{\hat{\beta }}_{a})\}\right]\;\;+P[(l\tilde{t}ad-1)S_{a}(t,X;{\hat{\beta }}_{a})]$$

(B21)
$$-P\left[\frac{\delta }{\pi_{\delta }(X;\;\hat{\eta })}\frac{A}{\pi_{A}(X;\;\hat{\rho })}\overset{\;t}{\underset{0\;}{\int }}\frac{d{\hat{M}}_{a}^{C}(u;\;{\hat{\gamma }}_{a})}{S_{a}^{C}(t,X;\;{\hat{\gamma }}_{a})}\left\{\frac{S_{a}(t,X)}{S_{a}(u,X)}-\frac{S_{a}(t,X;{\hat{\beta }}_{a})}{S_{a}(u,X;{\hat{\beta }}_{a})}\right\}\right],$$

for *a* ∈ {0, 1}. By Cauchy-Schwarz inequality and the positivity of *π*_*δ*_{*X*) and *π*_*A*_{*X*), we have

$$(B19)=P\left[\left\{\frac{\pi_{\delta }(X)\pi_{A}(X)-\pi_{\delta }(X;\;\hat{\eta })\pi_{A}(X;\;\hat{\rho })}{\pi_{\delta }(X;\;\hat{\eta })\pi_{A}(X;\;\hat{\rho })}\right\}\{S_{a}(t,X)-S_{a}(t,X;{\hat{\beta }}_{a})\}\right]\;\;≲\|\pi_{\delta }(X)\pi_{A}(X)-\pi_{\delta }(X;\;\hat{\eta })\pi_{A}(X;\;\hat{\rho })\|\cdot \|S_{a}(t,X)-S_{a}(t,X;{\hat{\beta }}_{a})\|\;\;\leq \{\|\pi_{\delta }(X)-\pi_{\delta }(X;\;\hat{\eta })\|+\|\pi_{A}(X)-\pi_{A}(X;\;\hat{\rho })\|\}\|S_{a}(t,X)-S_{a}(t,X;{\hat{\beta }}_{a})\|,$$

where ≲ indicates that the inequality holds up to a multiplicative constant. By iterated expectation, (B20) = 0. Lastly, by Cauchy-Schwarz inequality and iterated expectation, we have

$$(B21)\leq P\left[\frac{\pi_{\delta }(X)}{\pi_{\delta }(X;\;\hat{\eta })}\frac{\pi_{A}(X)}{\pi_{A}(X;\;\hat{\rho })}\overset{\;t}{\underset{0\;}{\int }}||\frac{dM_{a}^{C}(u;\;{\hat{\gamma }}_{a})}{S_{a}^{C}(t,X;\;{\hat{\gamma }}_{a})}||\cdot ||\frac{S_{a}(t,X)}{S_{a}(u,X)}-\frac{S_{a}(t,X;{\hat{\beta }}_{a})}{S_{a}(u,X;{\hat{\beta }}_{a})}||\right]\;\;≲P\left[\overset{\;t}{\underset{0\;}{\int }}\|d{\hat{M}}_{a}^{C}(u;\;{\hat{\gamma }}_{a})\|\cdot \;||\frac{S_{a}(t,X)}{S_{a}(u,X)}-\frac{S_{a}(t,X;{\hat{\beta }}_{a})}{S_{a}(u,X;{\hat{\beta }}_{a})}||\right],$$

where the last inequality holds by the condition (C2) in Theorem 3. Therefore, $P\{{𝒮}_{a}(t;\;\hat{\zeta })-{𝒮}_{a}(t;\;\zeta^{*})\}$ is bounded above by $\left\|\pi_{\delta }(X)-\pi_{\delta }(X;\;\hat{\eta })\|\cdot \|S_{a}(t,X)-S_{a}(t,X;{\hat{\beta }}_{a})\|\;+\;\|\pi_{A}(X)-\pi_{A}(X;\;\hat{\rho })\|\cdot \|S_{a}(t,X)-S_{a}(t,X;{\hat{\beta }}_{a})\|\;+P\int_{0}^{t}\|d{\hat{M}}_{a}^{C}(u;\;{\hat{\gamma }}_{a})\|\cdot \|S_{a}(u,X{)}^{-1}S_{a}(t,X)-S_{a}(u,X;{\hat{\beta }}_{a}{)}^{-1}S_{a}(t,X;{\hat{\beta }}_{a})\right\|$. It is negligible under the conditions (C1) and (C3) in Theorem 3; thus, $P\{\vartheta_{\tau }(\hat{\zeta })-\vartheta_{\tau }(\zeta^{*})\}$ is negligible as well and ${\hat{\theta }}_{\tau }^{\;{}^{\text{ACW}1}}$ achieves semiparametric efficiency. The proof for ${\hat{\theta }}_{\tau }^{\;{}^{\text{ACW}2}}$ is analogous by the small order difference between ${\hat{\theta }}_{\tau }^{\;{}^{\text{ACW}1}}$ and ${\hat{\theta }}_{\tau }^{\;{}^{\text{ACW}2}}$.
